# Association of noninvasive liver fibrosis scores with severity and outcomes among individuals with cardiovascular-kidney-metabolic syndrome: evidence from the NHANES 2005–2018

**DOI:** 10.1186/s12872-026-06148-2

**Published:** 2026-06-22

**Authors:** Yue Gao, Huiwen Chen, Jiawen Li, Mengli Chen, Haifeng Zhang

**Affiliations:** 1 Department of Geriatrics, Division of Cardiology, The First Affiliated Hospital with Nanjing Medical University, Nanjing, 210029 China; 2 Department of Geriatrics, Zhongda Hospital, School of Medicine, Southeast University, Nanjing, China; 3 Department of Cardiology, The Affiliated Suzhou Hospital of Nanjing Medical University, Suzhou Municipal Hospital, Gusu School, Nanjing Medical University, Suzhou, China

**Keywords:** Cardiovascular–kidney–metabolic syndrome, Liver fibrosis, Noninvasive liver fibrosis scores

## Abstract

**Background:**

This study aimed to investigate the associations of multiple noninvasive liver fibrosis scores with cardiovascular-kidney-metabolic (CKM) syndrome severity and clinical outcomes.

**Methods:**

This retrospective cohort study included 8,592 participants from the National Health and Nutrition Examination Survey 2005–2018. Six noninvasive liver fibrosis scores, including LiverRisk, metabolic dysfunction-associated fibrosis 5 (MAF-5), nonalcoholic fatty liver disease fibrosis score (NFS), Fibrosis-4 (FIB-4), steatosis-associated fibrosis estimator (SAFE), and aspartate aminotransferase-to-platelet ratio index (APRI), were evaluated. Logistic regression was used to assess associations with advanced CKM syndrome. Cox proportional hazards models and Fine–Gray competing-risk models were applied to evaluate associations with all-cause mortality and cardiovascular disease (CVD) mortality, respectively. Restricted cubic spline, subgroup, sensitivity, and incremental prediction analyses were also performed.

**Results:**

The participants had a mean age of 50.82 ± 0.22 years, 51.85% were female, and 12.30% had advanced CKM syndrome. During a median follow-up of 7.42 years, 599 all-cause deaths and 166 CVD deaths occurred. Higher LiverRisk, MAF-5, NFS, FIB-4, and SAFE scores were significantly associated with advanced CKM syndrome, whereas APRI was not. All six fibrosis scores were significantly associated with increased all-cause mortality. For CVD mortality, higher LiverRisk, NFS, FIB-4, and SAFE were significantly associated with increased risk, with corresponding sHRs of 3.84, 3.54, 2.25, and 3.63, respectively (all *P* < 0.05), whereas MAF-5 and APRI were not significantly associated with CVD mortality. These findings were supported by dose-response analyses and remained materially unchanged in sensitivity analyses.

**Conclusion:**

Higher LiverRisk, MAF-5, NFS, FIB-4, and SAFE scores were associated with advanced CKM syndrome, whereas all six scores were associated with all-cause mortality. LiverRisk, NFS, FIB-4, and SAFE showed more consistent associations with CVD mortality.

**Supplementary Information:**

The online version contains supplementary material available at https://doi.org/10.1186/s12872-026-06148-2.

## Introduction

Cardiovascular-kidney-metabolic (CKM) syndrome is a systemic condition characterized by the interconnection of cardiovascular disease (CVD), chronic kidney disease, and metabolic risk factors such as obesity and diabetes, which together drive progressive multi-organ damage and adverse health outcomes [1, 2]. The global burden of CKM-related diseases is rapidly increasing in parallel with the rising prevalence of metabolic risk factor [3]. Notably, almost 15% of U.S. adults met criteria for advanced CKM syndrome, which has been linked to a markedly elevated risk of all-cause mortality [2, 4]. Therefore, identifying markers that may help monitor disease progression, guide targeted intervention, and prevent further progression of CKM syndrome is of considerable clinical importance.

Steatotic liver disease is closely linked to cardiometabolic risk factors that drive disease development and progression [5]. Liver fibrosis is a major determinant of disease progression and clinical outcomes in patients with steatotic liver disease and other chronic liver diseases [6, 7]. Although liver disorders are not currently included in the CKM syndrome framework, emerging literature increasingly recognizes the liver as part of an interconnected cardio-kidney-liver-metabolic axis, supporting a potential link between liver fibrosis and CKM syndrome [8, 9]. Recent guidelines have emphasized that, although liver biopsy remains the reference standard, noninvasive tests have become practical tools for assessing liver fibrosis and stratifying risk in chronic liver disease [10]. Commonly used noninvasive fibrosis scores, including the LiverRisk score [11], metabolic dysfunction-associated fibrosis 5 (MAF-5) score [12], nonalcoholic fatty liver disease (NAFLD) fibrosis score (NFS) [13], Fibrosis-4 (FIB-4) score [14], steatosis-associated fibrosis estimator (SAFE) score [15], and aspartate aminotransferase (AST) to platelet ratio index (APRI) score [16], have been widely used as practical tools for fibrosis risk stratification in multiple studies.

Although previous studies have reported associations between liver fibrosis and CKM syndrome [17, 18], evidence remains limited regarding whether multiple commonly used noninvasive liver fibrosis scores are associated with CKM severity and subsequent clinical outcomes in the general population. Therefore, using data from the U.S. National Health and Nutrition Examination Survey (NHANES), the present study aimed to systematically evaluate the associations of six common noninvasive liver fibrosis scores with CKM severity and clinical outcomes.

## Methods

### Study population

We conducted a retrospective cohort study using NHANES data, a nationally representative survey with a complex, stratified, multistage probability design. The NHANES protocol was approved by the National Center for Health Statistics Research Ethics Review Board. Written informed consent was obtained from all participants or their guardians prior to data collection.

A total of 70,190 participants were initially identified for this study from 7 NHANES cycles (2005–2018). We excluded 61,598 individuals for the following reasons: (1) age < 30 or ≥ 80 years (*n* = 39,975) [19, 20]; (2) pregnant (*n* = 259); (3) missing key data for determining CKM syndrome stage (*n* = 18,511); (4) missing key data for assessing noninvasive liver fibrosis scores (*n* = 61); (5) missing data on key covariates or survival status (*n* = 2,792). The final study population for analysis consisted of 8,592 participants (Supplementary Figure S1). Furthermore, to conduct the multiple imputation sensitivity analysis, a broader cohort of 12,443 participants was established. This imputation cohort was derived by excluding only those aged < 30 or ≥ 80 years, pregnant individuals, those without fasting sample weights, and those with missing survival information. The baseline characteristics of the analyzed cohort (*n* = 8,592) and the excluded cohort (*n* = 3,851) are detailed and compared in Supplementary Table S3.

### CKM syndrome

According to the AHA Presidential Advisory on CKM syndrome and Aggarwal et al. [4], participants in this study were categorized into CKM syndrome stage 0–4. Further, stages 0–2 were determined as non-advanced CKM syndrome and stages 3–4 as advanced CKM syndrome. Details of the definition and classification of CKM syndrome and related conditions are provided in the Supplementary Methods. CKD stage was identified based on estimated glomerular filtration rate (eGFR) and the urine albumin-to-creatinine ratio (UACR) per Kidney Disease Improving Global Outcomes (KDIGO) criteria [21]. 10-year predicted CVD risk was estimated with the AHA Predicting Risk of CVD EVENTs (PREVENT) equations [19].

### Noninvasive liver fibrosis scores

Six noninvasive liver fibrosis scores—LiverRisk, MAF-5, NFS, FIB-4, SAFE, and APRI—were used to assess liver fibrosis. The detailed calculation formulas for these scores are described in the Supplementary Material. The liver fibrosis scores were categorized into distinct groups based on specific cut-off values according to previous studies [12, 13, 22, 23]. For LiverRisk: low risk, < 6, intermediate risk, 6 to 10, high risk, ≥ 10; For MAF-5: low risk, < 0, intermediate risk, 0 to 1, high risk, ≥ 1; For NFS: low risk, < -1.455, intermediate risk, -1.455 to 0.675, high risk, ≥ 0.675; For FIB-4: low risk, < 1.3/2, intermediate risk, 1.3/2 to 2.67, high risk, ≥ 2.67 (the cut-off of 1.3 for Age < 65, while the cut-off of 2 for Age ≥ 65); For SAFE: low risk, < 0, intermediate risk, 0 to 100, high risk, ≥ 100; For APRI: low risk, < 1, high risk, ≥ 1.

### Assessment of outcomes

The outcomes of this study included all-cause mortality and CVD mortality. CVD mortality was defined as death attributable to heart diseases (ICD-10 classifications I00-I09, I11, I13, and I20–I51) and cerebrovascular diseases (ICD-10 classifications I60–I69). Mortality data were obtained from the NHANES-linked National Death Index (NDI), which provides detailed, validated, and reliable information on mortality status and causes of death for NHANES participants.

### Assessment of covariates

Demographic characteristics, socioeconomic factors, lifestyle factors, physical examination, laboratory tests, and comorbid conditions were collected. Demographic characteristics included age, sex (male or female), race/ethnicity categories (Mexican American, non-Hispanic black, non-Hispanic white, or other), education level (below high school, high school graduate, and above high school) and marital status (married/living with a partner or other). The family poverty income ratio (PIR), a socioeconomic status indicator, was defined as the ratio of family income to the federal poverty level and categorized into two groups: < 3, and ≥ 3.0. Smoking status was categorized into three groups: never, former, or current, and drinking status was classified as nondrinker, low-to-moderate drinker (≤ 2 drinks/day for men and ≤ 1 drink/day for women), and heavy drinker (> 2 drinks/day for men and > 1 drink/day for women). Physical activity was stratified by intensity into three groups: less than moderate, moderate, and vigorous. Physical examination data included body mass index (BMI), waist circumference (WC), systolic blood pressure (SBP) and diastolic blood pressure (DBP). A range of clinical laboratory markers were assessed, including platelets (PLT), albumin (ALB), globulin (GLB), fasting blood glucose (FBG), glycated haemoglobin (HbA1c), alanine aminotransferase (ALT), AST, gamma-glutamyl transferase (GGT), total cholesterol (TC), high-density lipoprotein (HDL) cholesterol, low-density lipoprotein (LDL) cholesterol, triglyceride (TG), blood urea nitrogen (BUN), serum creatinine (Scr), uric acid (UA), UACR. eGFR was calculated with the CKD-EPI equation. Hypertension was defined as self-reported diagnosis of hypertension, use of anti-hypertensive medications, or systolic blood pressure ≥ 130 mmHg or diastolic blood pressure ≥ 80 mmHg. Dyslipidemia was defined by any of the following criteria: total cholesterol > 200 mg/dL, triglycerides > 150 mg/dL, LDL cholesterol > 130 mg/dL, or low HDL cholesterol (< 40 mg/dL for male and < 50 mg/dL for female), or use of anti-dyslipidemia medication. Diabetes was identified based on self-reported diagnosis of diabetes, use of insulin or oral hypoglycaemic agents, or fasting blood glucose ≥ 125 mg/dL, or HbA1c ≥ 6.5%. NAFLD was determined as hepatic steatosis (United States fatty liver index > 30) in the absence of excessive alcohol consumption or other chronic liver disease.

### Statistical analysis

Following the National Center for Health Statistics guidelines, all analyses were conducted using NHANES sample weights, strata, and primary sampling units to ensure nationally representative estimates. Categorical variables were summarized as weighted percentages, while continuous variables were expressed as the means and standard errors. For group comparisons, Mann–Whitney U tests or analysis of variance (ANOVA) were used for continuous variables, and Chi-square tests were used for categorical variables.

To evaluate the associations between the LiverRisk, MAF-5, NFS, FIB-4, SAFE, APRI scores and advanced CKM syndrome, we used multivariable logistic regression. The results were expressed as odds ratios (ORs) with 95% confidence intervals (CIs). Two models were constructed for this analysis: Model 1 was unadjusted, and Model 2 was adjusted for age, sex, race/ethnicity, education level, PIR, marital status, smoking status, drinking status, BMI, physical activity, and NAFLD [24, 25].

Kaplan–Meier curves were generated for all-cause mortality. For CVD mortality, cumulative incidence curves were constructed with non-CVD death treated as a competing event. Further, we applied Cox proportional hazards regression models to assess the associations between the LiverRisk, MAF-5, NFS, FIB-4, SAFE, and APRI scores and all-cause mortality. For CVD mortality, Fine-Gray competing risk regression models were employed to compute subdistribution hazard ratios, considering non-CVD death as a competing event. The proportional hazards assumption was evaluated using Schoenfeld residuals, and for any violations, time-dependent covariates were incorporated into the models. Multicollinearity among covariates was examined using the variance inflation factor (VIF), with values less than 10 indicating an acceptable level of multicollinearity. Three sequentially adjusted multivariable models were constructed to account for potential confounders [26, 27]. Model 1 was unadjusted. Model 2 was adjusted for age, sex, race/ethnicity, education level, PIR, marital status, smoking status, drinking status, and physical activity. Model 3 further incorporated Model 2 variables plus BMI, eGFR, hypertension, diabetes, hyperlipidaemia, and NAFLD. Importantly, to prevent collinearity and over-adjustment, any baseline variables inherently used in the calculation of a specific fibrosis score were explicitly excluded from the corresponding multivariable models (Supplementary Table S1). We also used restricted cubic spline (RCS) regression models with 3 knots to determine the dose-response relationships between LiverRisk, MAF-5, NFS, FIB-4, SAFE, APRI scores and mortality outcomes. To investigate the incremental prognostic value of noninvasive liver fibrosis, each fibrosis score was added individually to the base PREVENT model. The predictive performance of the updated models for 10-year mortality risk was assessed and compared using receiver operating characteristic (ROC) curves and the corresponding area under the curve (AUC) at 10 years.

To further evaluate the robustness of the findings, subgroup analyses were conducted across various strata, including sex, age group, race/ethnicity, and advanced CKM syndrome. In addition, we conducted three sensitivity analyses to assess the robustness of the results. First, we repeated the primary analyses using multiple imputation to handle missing data for baseline covariates. Second, we excluded participants whose underlying cause of death was attributed to accidents. Third, we excluded participants who died within the first two years of follow-up to minimize potential bias from early mortality and reverse causation. R software (version 4.3.2) was used for all statistical analyses, and a two-tailed *P* < 0.05 was considered statistically significant.

## Results

### Baseline characteristics

**Fig. 1:**
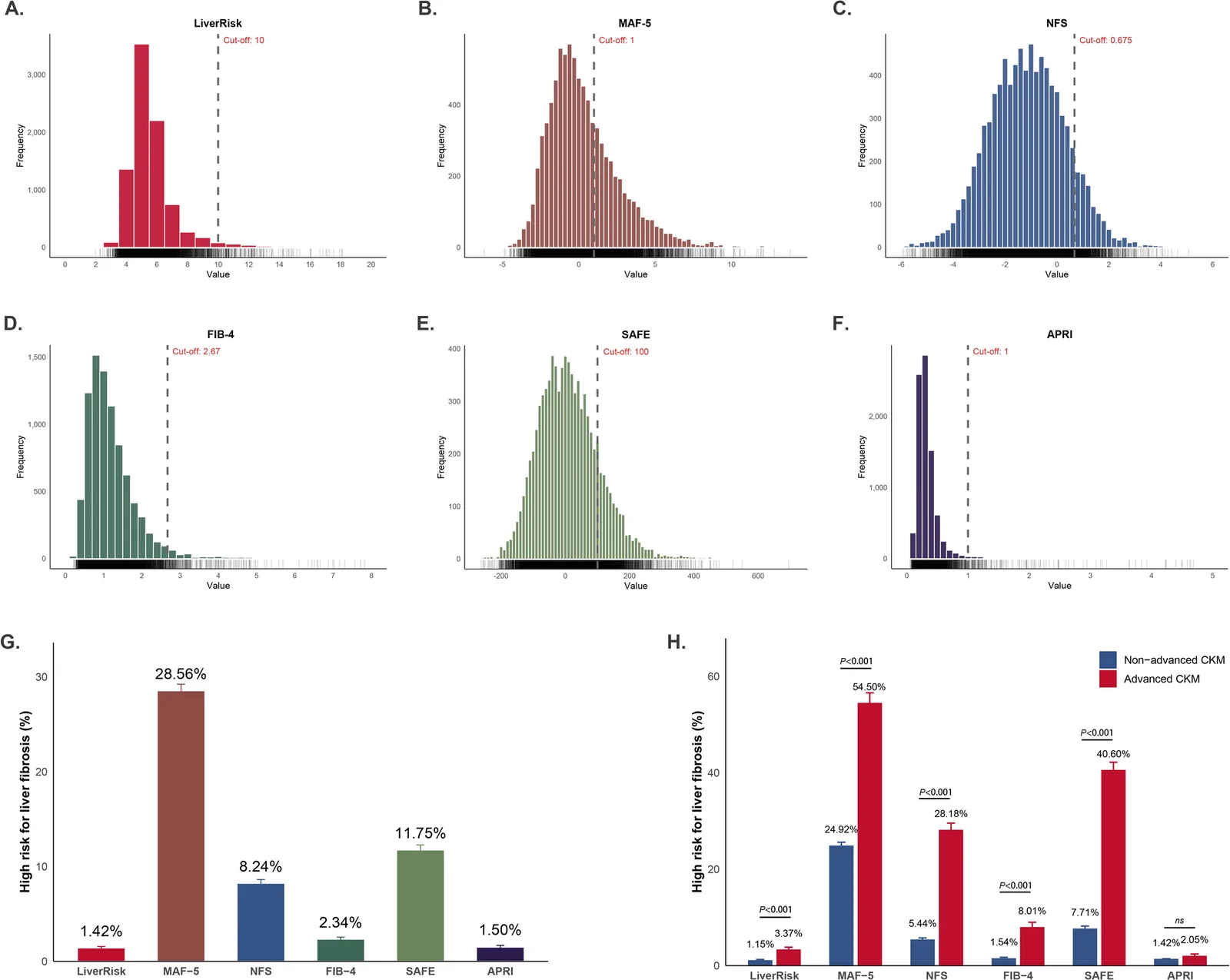
Prevalence of high risk for liver fibrosis as determined by noninvasive fibrosis scores. **A**–**F**, Overall distribution of noninvasive liver fibrosis scores; **G**, Weighted percentage of high risk for liver fibrosis in the general population based on different liver fibrosis scores. **H**, Weighted percentage of high risk for liver fibrosis in non-advanced CKM syndrome and advanced CKM syndrome. APRI, aspartate aminotransferase to platelet ratio index score; CKM, cardiovascular-kidney-metabolic; FIB-4, Fibrosis-4 score; LiverRisk, LiverRisk score; MAF-5, metabolic dysfunction-associated fibrosis 5 score; NFS, nonalcoholic fatty liver disease fibrosis score; SAFE, steatosis-associated fibrosis estimator score

Table 1 summarizes the baseline characteristics of the study population. Of the 8,592 participants (4,410 women and 4,182 men; with a mean age of 50.82 ± 0.22 years), 8.19% met criteria for CKM stage 0, 21.56% for CKM stage 1, 57.95% for CKM stage 2, 4.12% for CKM stage 3, and 8.18% for CKM stage 4. Figure 1 illustrates the distribution of different noninvasive liver fibrosis scores. The prevalence of high liver fibrosis risk was 1.42%, 28.56%, 8.24%, 2.34%, 11.75%, 1.50% as determined by LiverRisk, MAF-5, NFS, FIB-4, SAFE and APRI scores, respectively (Supplementary Table S2 and Fig. 1). Notably, the proportions of high liver fibrosis risk determined by all liver fibrosis scores except the APRI score were significantly higher in participants with advanced CKM stage compared with those with non-advanced CKM stage (Supplementary Table S2 and Fig. 1).

**Table 1:** Baseline characteristics of the study population stratified by CKM stages

Characteristic	Overall, *n* = 8,592 (100.00%)	CKM 0, *n* = 561 (8.19%)	CKM 1, *n* = 1,632 (21.56%)	CKM 2, *n* = 5,012 (57.95%)	CKM 3, *n* = 538 (4.12%)	CKM 4, *n* = 849 (8.18%)	*P*
Age	50.82 ± 0.22	43.31 ± 0.50	45.95 ± 0.38	50.72 ± 0.25	70.89 ± 0.39	61.75 ± 0.43	< 0.001
Sex							< 0.001
Female	4,410 (51.85%)	393 (72.10%)	905 (53.85%)	2,523 (49.93%)	221 (43.94%)	368 (43.83%)	
Male	4,182 (48.15%)	168 (27.90%)	727 (46.15%)	2,489 (50.07%)	317 (56.06%)	481 (56.17%)	
Race/ethnicity							< 0.001
Mexican American	1,316 (7.27%)	44 (3.16%)	273 (8.27%)	844 (8.02%)	68 (5.25%)	87 (4.43%)	
Non-Hispanic Black	1,720 (10.24%)	60 (5.52%)	288 (9.23%)	1,035 (10.72%)	118 (11.94%)	219 (13.42%)	
Non-Hispanic White	3,781 (70.73%)	314 (79.66%)	729 (71.34%)	2,074 (69.07%)	252 (71.81%)	412 (71.35%)	
Other	1,775 (11.76%)	143 (11.66%)	342 (11.16%)	1,059 (12.19%)	100 (11.00%)	131 (10.80%)	
Education level							< 0.001
Under high school	1,892 (13.91%)	72 (8.18%)	298 (11.07%)	1,127 (14.37%)	150 (19.25%)	245 (21.22%)	
High school or equivalent	1,936 (22.39%)	92 (14.05%)	295 (17.73%)	1,187 (24.19%)	146 (28.12%)	216 (27.33%)	
Above high school	4,764 (63.70%)	397 (77.77%)	1,039 (71.19%)	2,698 (61.44%)	242 (52.63%)	388 (51.45%)	
Family PIR							< 0.001
< 3	4,964 (43.88%)	236 (28.92%)	843 (37.99%)	2,919 (44.91%)	365 (58.53%)	601 (59.71%)	
≥ 3	3,628 (56.12%)	325 (71.08%)	789 (62.01%)	2,093 (55.09%)	173 (41.47%)	248 (40.29%)	
Marital status							< 0.001
Married or living with a partner	4,827 (59.35%)	357 (67.24%)	1,000 (63.51%)	2,780 (57.69%)	281 (53.75%)	409 (55.02%)	
Other	3,765 (40.65%)	204 (32.76%)	632 (36.49%)	2,232 (42.31%)	257 (46.25%)	440 (44.98%)	
Smoking status							< 0.001
Never smoker	4,718 (54.79%)	356 (63.08%)	999 (60.33%)	2,795 (54.75%)	231 (42.76%)	337 (38.25%)	
Former smoker	2,177 (26.40%)	89 (16.15%)	359 (24.49%)	1,219 (25.92%)	208 (40.78%)	302 (37.75%)	
Current smoker	1,697 (18.82%)	116 (20.77%)	274 (15.18%)	998 (19.33%)	99 (16.45%)	210 (24.00%)	
Drinking status							< 0.001
Nondrinker	2,693 (24.71%)	140 (18.17%)	434 (20.54%)	1,581 (25.24%)	223 (39.45%)	315 (31.11%)	
Low-to-moderate drinker	5,066 (63.97%)	364 (69.90%)	1,054 (68.98%)	2,922 (63.09%)	273 (51.55%)	453 (57.35%)	
Heavy drinker	833 (11.32%)	57 (11.93%)	144 (10.49%)	509 (11.67%)	42 (9.00%)	81 (11.54%)	
Physical activity							< 0.001
Less than moderate	3,536 (37.29%)	188 (29.04%)	548 (31.18%)	2,063 (38.06%)	303 (53.33%)	434 (48.10%)	
Moderate	2,708 (34.66%)	220 (42.81%)	555 (37.35%)	1,563 (34.04%)	136 (26.28%)	234 (27.94%)	
Vigorous	2,348 (28.05%)	153 (28.16%)	529 (31.46%)	1,386 (27.90%)	99 (20.39%)	181 (23.96%)	
BMI	29.37 ± 0.12	22.01 ± 0.10	28.16 ± 0.17	30.54 ± 0.14	30.52 ± 0.34	31.04 ± 0.31	< 0.001
SBP	122.33 ± 0.27	107.60 ± 0.56	112.48 ± 0.29	125.83 ± 0.28	140.36 ± 1.28	129.22 ± 0.83	< 0.001
DBP	71.60 ± 0.20	65.54 ± 0.47	67.98 ± 0.22	74.24 ± 0.23	68.65 ± 0.76	69.98 ± 0.66	< 0.001
Platelets	244.94 ± 0.93	237.83 ± 3.12	241.65 ± 1.88	250.16 ± 1.28	234.93 ± 3.27	228.71 ± 3.13	< 0.001
BUN	13.55 ± 0.09	12.29 ± 0.21	12.92 ± 0.12	13.28 ± 0.09	18.56 ± 0.47	15.86 ± 0.31	< 0.001
Scr	0.88 ± 0.00	0.82 ± 0.01	0.85 ± 0.01	0.86 ± 0.00	1.18 ± 0.05	1.00 ± 0.02	< 0.001
UA	5.49 ± 0.02	4.53 ± 0.06	5.14 ± 0.04	5.66 ± 0.03	6.16 ± 0.09	5.87 ± 0.08	< 0.001
ALT	25.60 ± 0.23	20.09 ± 0.65	23.04 ± 0.38	27.52 ± 0.35	24.46 ± 1.19	24.83 ± 0.66	< 0.001
AST	25.34 ± 0.24	23.21 ± 0.58	23.63 ± 0.30	26.10 ± 0.35	25.75 ± 0.89	26.43 ± 0.73	< 0.001
GGT	29.01 ± 0.56	16.73 ± 0.59	21.97 ± 0.60	32.40 ± 0.86	30.91 ± 1.73	34.88 ± 1.86	< 0.001
HbA1c	5.65 ± 0.01	5.18 ± 0.01	5.34 ± 0.01	5.72 ± 0.02	6.38 ± 0.06	6.06 ± 0.05	< 0.001
FBG	107.38 ± 0.41	90.31 ± 0.27	98.74 ± 0.31	109.71 ± 0.51	129.24 ± 1.94	119.70 ± 1.64	< 0.001
TG	121.05 ± 1.02	69.83 ± 1.26	81.14 ± 0.79	140.15 ± 1.47	138.77 ± 4.05	133.25 ± 3.42	< 0.001
LDL-c	117.59 ± 0.58	106.66 ± 1.64	118.56 ± 1.00	121.65 ± 0.74	107.03 ± 2.11	102.53 ± 1.49	< 0.001
HDL-c	55.09 ± 0.25	67.21 ± 0.92	59.36 ± 0.50	52.50 ± 0.29	50.95 ± 0.84	52.18 ± 0.95	< 0.001
TC	196.89 ± 0.69	187.82 ± 1.90	194.15 ± 1.16	202.18 ± 0.89	185.74 ± 2.26	181.36 ± 1.65	< 0.001
UACR	28.93 ± 2.41	7.33 ± 0.28	6.45 ± 0.14	21.78 ± 1.53	208.40 ± 44.99	69.97 ± 13.99	< 0.001
eGFR	91.55 ± 0.32	96.65 ± 0.79	95.82 ± 0.58	92.82 ± 0.40	66.22 ± 1.17	79.01 ± 0.89	< 0.001
Diabetes							< 0.001
No	6,874 (85.14%)	561 (100.00%)	1,632 (100.00%)	3,979 (83.72%)	204 (39.11%)	498 (64.37%)	
Yes	1,718 (14.86%)	0 (0.00%)	0 (0.00%)	1,033 (16.28%)	334 (60.89%)	351 (35.63%)	
Hypertension							< 0.001
No	3,701 (47.48%)	561 (100.00%)	1,632 (100.00%)	1,340 (27.61%)	29 (5.52%)	139 (18.52%)	
Yes	4,891 (52.52%)	0 (0.00%)	0 (0.00%)	3,672 (72.39%)	509 (94.48%)	710 (81.48%)	
NAFLD							< 0.001
No	5,744 (68.51%)	560 (99.79%)	1,417 (87.69%)	3,063 (60.92%)	266 (47.61%)	438 (50.97%)	
Yes	2,848 (31.49%)	1 (0.21%)	215 (12.31%)	1,949 (39.08%)	272 (52.39%)	411 (49.03%)	
LiverRisk	5.45 ± 0.02	4.70 ± 0.03	5.03 ± 0.02	5.51 ± 0.03	6.52 ± 0.08	6.29 ± 0.07	< 0.001
MAF-5	0.17 ± 0.04	-1.96 ± 0.05	-0.70 ± 0.05	0.50 ± 0.04	1.79 ± 0.14	1.45 ± 0.10	< 0.001
NFS	-1.30 ± 0.02	-2.72 ± 0.05	-1.63 ± 0.04	-1.23 ± 0.03	0.12 ± 0.06	-0.19 ± 0.06	< 0.001
FIB-4	1.16 ± 0.01	1.03 ± 0.03	1.03 ± 0.02	1.12 ± 0.02	1.80 ± 0.05	1.59 ± 0.04	< 0.001
SAFE	-5.33 ± 1.49	-81.27 ± 3.67	-40.92 ± 1.90	0.12 ± 1.66	108.69 ± 4.34	68.27 ± 3.81	< 0.001
APRI	0.35 ± 0.01	0.32 ± 0.01	0.32 ± 0.00	0.35 ± 0.01	0.38 ± 0.02	0.39 ± 0.02	< 0.001

Continuous variables are presented as weighted mean ± SE. Categorical variables are presented as unweighted *n* (weighted %)

*ALT* Alanine aminotransferase, *APRI* Aspartate aminotransferase to platelet ratio index score, *AST* Aspartate aminotransferase, *BMI* Body mass index, *BUN* Blood urea nitrogen, *CKM* Cardiovascular-kidney-metabolic, *DBP* Diastolic blood pressure, *eGFR* Estimated glomerular filtration rate, *FBG* Fasting blood glucose, *FIB-4* Fibrosis-4 score, *GGT* Gamma-glutamyl transferase, *HbA1c* Haemoglobin A1c, *HDL-c* High-density lipoprotein cholesterol, *LDL-c* Low-density lipoprotein cholesterol, *LiverRisk* LiverRisk score, *MAF-5* Metabolic dysfunction-associated fibrosis-5 score, *NAFLD* Nonalcoholic fatty liver disease, *NFS* Nonalcoholic fatty liver disease fibrosis score, *PIR* Poverty income ratio, *SAFE* Steatosis-associated fibrosis estimator score, *SBP* Systolic blood pressure, *SE* Standard error, *Scr* Serum creatinine, *TC* Total cholesterol, *TG* Triglyceride, *UA* Uric acid, and *UACR* Urine albumin-to-creatinine ratio

### Association between liver fibrosis and advanced CKM syndrome

When analyzed as continuous variables, all six noninvasive liver fibrosis scores were significantly associated with the presence of advanced CKM syndrome in Model 1 (all *P* < 0.05). However, after adjusting for covariates in Model 2, these significant associations persisted for all scores (all *P* < 0.001) except for APRI (*P* = 0.243) (Table 2).

**Table 2:** Association between noninvasive liver fibrosis scores and advanced CKM syndrome

Noninvasive liver fibrosis scores	Advanced CKM syndrome cases, *n* (%)	Model 1		Model 2	
		OR (95% CI)	*P*	OR (95% CI)	*P*
Continuous variables (per 1 SD increase)					
LiverRisk	1387 (12.31)	1.78 (1.63, 1.95)	< 0.001	1.62 (1.48, 1.78)	< 0.001
MAF-5	1387 (12.31)	1.81 (1.69, 1.95)	< 0.001	1.72 (1.56, 1.91)	< 0.001
NFS	1387 (12.31)	2.96 (2.73, 3.21)	< 0.001	2.71 (2.47, 2.98)	< 0.001
FIB-4	1387 (12.31)	1.89 (1.52, 2.34)	< 0.001	1.86 (1.46, 2.38)	< 0.001
SAFE	1387 (12.31)	2.94 (2.64, 3.29)	< 0.001	2.94 (2.55, 3.39)	< 0.001
APRI	1387 (12.31)	1.08 (1.02, 1.15)	0.006	1.05 (0.96, 1.15)	0.243
Categorical variables					
LiverRisk					
Low	623 (7.32)	Reference		Reference	
Intermediate	704 (30.33)	5.51 (4.70, 6.46)	< 0.001	4.63 (3.82, 5.62)	< 0.001
High	60 (29.13)	5.20 (3.59, 7.54)	< 0.001	3.96 (2.70, 5.81)	< 0.001
MAF-5					
Low	374 (6.46)	Reference		Reference	
Intermediate	226 (12.52)	2.07 (1.61, 2.66)	< 0.001	1.55 (1.14, 2.10)	0.005
High	787 (23.48)	4.44 (3.62, 5.45)	< 0.001	2.99 (2.35, 3.79)	< 0.001
NFS					
Low	189 (4.02)	Reference		Reference	
Intermediate	771 (15.34)	4.32 (3.46, 5.41)	< 0.001	3.76 (3.00, 4.73)	< 0.001
High	427 (42.11)	17.36 (13.59, 22.17)	< 0.001	12.96 (9.83, 17.08)	< 0.001
FIB-4					
Low	908 (10.29)	Reference		Reference	
Intermediate	352 (17.07)	1.79 (1.48, 2.18)	< 0.001	1.88 (1.52, 2.32)	< 0.001
High	127 (42.14)	6.35 (4.37, 9.22)	< 0.001	6.31 (4.22, 9.43)	< 0.001
SAFE					
Low	182 (3.63)	Reference		Reference	
Intermediate	570 (16.45)	5.23 (4.08, 6.69)	< 0.001	5.24 (4.10, 6.69)	< 0.001
High	635 (42.51)	19.64 (15.48, 24.92)	< 0.001	18.95 (14.51, 24.73)	< 0.001
APRI					
Low	1349 (12.24)	Reference		Reference	
High	38 (16.83)	1.45 (0.93, 2.25)	0.096	1.18 (0.71, 1.97)	0.522

Model 1: Unadjusted

Model 2: Adjusted for age, sex, race/ethnicity, education, family PIR, marital status, smoking, drinking, BMI, physical activity, and NAFLD. Variables used to derive each score were excluded from Model 2, as described in the Methods

*APRI* Aspartate aminotransferase to platelet ratio index score, *BMI* Body mass index, *CI* Confidence interval, *CKM* Cardiovascular-kidney-metabolic, *FIB-4* Fibrosis-4 score, *LiverRisk* LiverRisk score, *MAF-5* Metabolic dysfunction-associated fibrosis-5 score, *NAFLD* Nonalcoholic fatty liver disease, *NFS* Nonalcoholic fatty liver disease fibrosis score, *OR* Odds ratio, *PIR* Poverty income ratio, *SAFE* steatosis-associated fibrosis estimator score, and *SD* Standard deviation

As categorical variables, participants with high risk of liver fibrosis determined by LiverRisk (OR = 3.96, 95% CI: 2.70–5.81), MAF-5 (OR = 2.99, 95% CI: 2.35–3.79), NFS (OR = 12.96, 95% CI: 9.83–17.08), FIB-4 (OR = 6.31, 95% CI: 4.22–9.43), or SAFE (OR = 18.95, 95% CI: 14.51–24.73) had significantly higher odds of advanced CKM syndrome compared to those with low risk (all *P* < 0.001, Table 2). However, high risk of liver fibrosis identified by APRI (OR = 1.18, 95% CI: 0.71–1.97, *P* = 0.522) was not significantly associated with advanced CKM syndrome (Table 2).

### Association between liver fibrosis and mortality outcomes

**Fig. 2:**
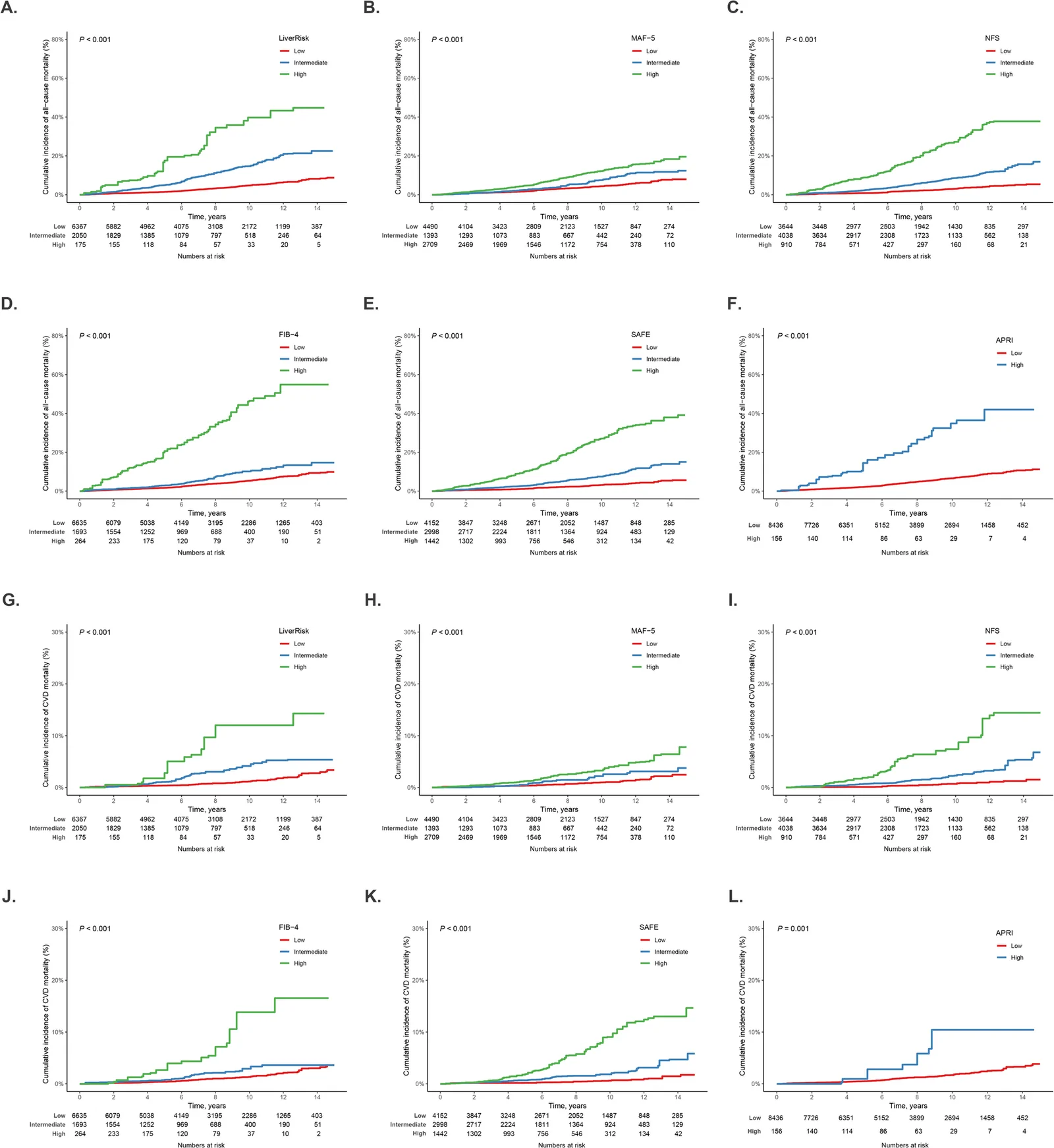
Kaplan–Meier curves for all-cause mortality and cumulative incidence curves for CVD mortality across different liver fibrosis risk groups. **A**–**F**, Kaplan–Meier curves for all-cause mortality according to LiverRisk **A**, MAF-5 **B**, NFS **C**, FIB-4 **D**, SAFE **E**, and APRI **F** risk groups. **G**–**L**, cumulative incidence curves for CVD mortality according to LiverRisk **G**, MAF-5 **H**, NFS **I**, FIB-4 **J**, SAFE **K**, and APRI **L** risk groups. APRI, aspartate aminotransferase to platelet ratio index score; FIB-4, Fibrosis-4 score; LiverRisk, LiverRisk score; MAF-5, metabolic dysfunction-associated fibrosis 5 score; NFS, nonalcoholic fatty liver disease fibrosis score; SAFE, steatosis-associated fibrosis estimator score

During a median follow-up of 7.42 years (interquartile range, 4.08–10.83), 599 deaths were recorded, with 166 attributed to CVD. Figure 2 shows Kaplan–Meier curves for all-cause mortality and cumulative incidence curves for CVD mortality across different liver fibrosis groups. Compared to those at low risk, participants with a high risk of liver fibrosis showed significantly higher cumulative incidence of both all-cause mortality and CVD mortality (all *P* < 0.05).

Analyzed as categorical variables in the Cox proportional hazards analyses, high liver fibrosis risk, as determined by all six liver fibrosis scores, was significantly associated with an increased risk of all-cause mortality compared with low liver fibrosis risk: LiverRisk (HR = 6.66, 95% CI: 4.12–10.76), MAF-5 (HR = 1.66, 95% CI: 1.23–2.24), NFS (HR = 5.48, 95% CI: 3.82–7.85), FIB-4 (HR = 5.73, 95% CI: 4.08–8.07), SAFE (HR = 5.20, 95% CI: 3.64–7.44), and APRI (HR = 3.64, 95% CI: 2.27–5.84) (all *P* < 0.001; all *P* for trend < 0.001, Table 3). Similarly, when analyzed as continuous variables in the fully adjusted models, all six noninvasive liver fibrosis scores remained significantly associated with an increased risk of all-cause mortality (all *P* < 0.001, Table 3).

**Table 3:** Associations between noninvasive liver fibrosis scores and all-cause mortality among participants with CKM syndrome

Noninvasive liver fibrosis scores	Event, *n* (%)	Model 1		Model 2		Model 3	
		HR (95% CI)	*P*	HR (95% CI)	*P*	HR (95% CI)	*P*
Continuous variables (per 1 SD increase)							
LiverRisk	599 (5.35)	1.30 (1.23, 1.39)	< 0.001	1.26 (1.19, 1.33)	< 0.001	1.25 (1.18, 1.33)	< 0.001
MAF-5	599 (5.35)	1.51 (1.39, 1.65)	< 0.001	1.30 (1.17, 1.46)	< 0.001	1.31 (1.14, 1.51)	< 0.001
NFS	599 (5.35)	2.20 (1.95, 2.49)	< 0.001	2.04 (1.80, 2.31)	< 0.001	1.81 (1.57, 2.08)	< 0.001
FIB-4	599 (5.35)	1.31 (1.25, 1.37)	< 0.001	1.27 (1.20, 1.33)	< 0.001	1.23 (1.16, 1.29)	< 0.001
SAFE	599 (5.35)	2.07 (1.90, 2.27)	< 0.001	1.92 (1.76, 2.11)	< 0.001	1.78 (1.60, 1.98)	< 0.001
APRI	599 (5.35)	1.14 (1.10, 1.18)	< 0.001	1.13 (1.10, 1.17)	< 0.001	1.13 (1.09, 1.16)	< 0.001
Categorical variables							
LiverRisk							
Low	324 (3.83)	Reference		Reference		Reference	
Intermediate	238 (9.80)	3.27 (2.65, 4.04)	< 0.001	2.92 (2.32, 3.67)	< 0.001	2.27 (1.77, 2.91)	< 0.001
High	37 (25.03)	9.29 (5.49, 15.73)	< 0.001	7.64 (4.68, 12.47)	< 0.001	6.66 (4.12, 10.76)	< 0.001
*P* for trend		< 0.001		< 0.001		< 0.001	
MAF-5							
Low	213 (3.67)	Reference		Reference		Reference	
Intermediate	104 (5.57)	1.61 (1.23, 2.10)	0.001	1.18 (0.90, 1.55)	0.218	1.20 (0.90, 1.61)	0.219
High	282 (8.46)	2.68 (2.16, 3.32)	< 0.001	1.65 (1.31, 2.08)	< 0.001	1.66 (1.23, 2.24)	< 0.001
*P* for trend		< 0.001		< 0.001		< 0.001	
NFS							
Low	122 (2.74)	Reference		Reference		Reference	
Intermediate	314 (5.95)	2.84 (2.17, 3.72)	< 0.001	2.64 (1.99, 3.51)	< 0.001	2.17 (1.62, 2.90)	< 0.001
High	163 (16.60)	10.13 (7.27, 14.10)	< 0.001	7.97 (5.80, 10.95)	< 0.001	5.48 (3.82, 7.85)	< 0.001
*P* for trend		< 0.001		< 0.001		< 0.001	
FIB-4							
Low	369 (4.37)	Reference		Reference		Reference	
Intermediate	150 (6.45)	1.77 (1.40, 2.25)	< 0.001	1.70 (1.33, 2.18)	< 0.001	1.41 (1.11, 1.79)	0.005
High	80 (29.47)	10.77 (7.59, 15.26)	< 0.001	8.64 (6.12, 12.19)	< 0.001	5.73 (4.08, 8.07)	< 0.001
*P* for trend		< 0.001		< 0.001		< 0.001	
SAFE							
Low	119 (2.58)	Reference		Reference		Reference	
Intermediate	219 (5.90)	2.59 (1.95, 3.44)	< 0.001	2.35 (1.75, 3.15)	< 0.001	1.96 (1.44, 2.66)	< 0.001
High	261 (17.05)	9.23 (6.87, 12.40)	< 0.001	7.19 (5.29, 9.76)	< 0.001	5.20 (3.64, 7.44)	< 0.001
*P* for trend		< 0.001		< 0.001		< 0.001	
APRI							
Low	564 (5.08)	Reference		Reference		Reference	
High	35 (22.52)	5.76 (3.57, 9.30)	< 0.001	4.07 (2.48, 6.65)	< 0.001	3.64 (2.27, 5.84)	< 0.001

Model 1: Unadjusted

Model 2: Adjusted for age, sex, race/ethnicity, education level, family PIR, marital status, smoking, drinking, and physical activity

Model 3: Adjusted for Model 2 variables plus BMI, eGFR, hypertension, diabetes, hyperlipidemia, and NAFLD

For each fibrosis score, variables used in the calculation of that score were not additionally included in the corresponding multivariable model, as described in the Methods

*APRI* Aspartate aminotransferase to platelet ratio index score, *BMI* Body mass index, *CI* Confidence interval, *CKM* Cardiovascular-kidney-metabolic, *eGFR* Estimated glomerular filtration rate, *FIB-4* Fibrosis-4 index score, *LiverRisk* LiverRisk score, *MAF-5* Metabolic dysfunction-associated fibrosis-5 score, *NAFLD* Nonalcoholic fatty liver disease, *NFS* Nonalcoholic fatty liver disease fibrosis score, *PIR* Poverty income ratio, *SAFE* Steatosis-associated fibrosis estimator score, *SD* Standard deviation, *HR* Hazard ratio

With regard to CVD mortality, Fine-Gray competing risk regression models were employed. Compared with those at low risk, participants with a high risk of liver fibrosis had a significantly increased risk of CVD mortality when assessed by LiverRisk (sHR = 3.84, 95% CI: 1.54–9.56), NFS (sHR = 3.54, 95% CI: 1.65–7.60), FIB-4 (sHR = 2.25, 95% CI: 1.17–4.33), and SAFE (sHR = 3.63, 95% CI: 1.77–7.44). In contrast, high risk defined by MAF-5 or APRI was not significantly associated with CVD mortality (Table 4). Consistent with the categorical analyses, continuous evaluations of the LiverRisk, NFS, FIB-4, and SAFE scores demonstrated significant associations with increased CVD mortality in the fully adjusted models, with the exceptions of MAF-5 and APRI (Table 4).

**Table 4:** Associations between noninvasive liver fibrosis scores and CVD mortality among participants with CKM syndrome

Noninvasive liver fibrosis scores	Event, *n* (%)	Model 1		Model 2		Model 3	
		sHR (95% CI)	*P*	sHR (95% CI)	*P*	sHR (95% CI)	*P*
Continuous variables (per 1 SD increase)							
LiverRisk	166 (1.43)	1.25 (1.16, 1.35)	< 0.001	1.22 (1.14, 1.30)	< 0.001	1.20 (1.10, 1.31)	< 0.001
MAF-5	166 (1.43)	1.51 (1.32, 1.73)	< 0.001	1.20 (1.01, 1.43)	0.039	1.11 (0.86, 1.43)	0.413
NFS	166 (1.43)	2.31 (1.88, 2.83)	< 0.001	1.99 (1.64, 2.41)	< 0.001	1.63 (1.31, 2.02)	< 0.001
FIB-4	166 (1.43)	1.23 (1.14, 1.33)	< 0.001	1.19 (1.11, 1.28)	< 0.001	1.14 (1.04, 1.24)	0.004
SAFE	166 (1.43)	1.99 (1.79, 2.22)	< 0.001	1.76 (1.56, 1.98)	< 0.001	1.51 (1.28, 1.77)	< 0.001
APRI	166 (1.43)	1.10 (1.04, 1.16)	0.001	1.06 (0.97, 1.16)	0.188	1.05 (0.96, 1.16)	0.275
Categorical variables							
LiverRisk							
Low	94 (1.10)	Reference		Reference		Reference	
Intermediate	60 (2.37)	2.70 (1.86, 3.92)	< 0.001	2.31 (1.58, 3.38)	< 0.001	1.52 (0.88, 2.62)	0.133
High	12 (6.01)	6.68 (3.24, 13.77)	< 0.001	4.85 (2.29, 10.26)	< 0.001	3.84 (1.54, 9.56)	0.004
*P* for trend		< 0.001		< 0.001		0.021	
MAF-5							
Low	58 (0.89)	Reference		Reference		Reference	
Intermediate	26 (1.47)	1.74 (0.98, 3.12)	0.060	1.08 (0.59, 1.98)	0.797	1.01 (0.49, 2.08)	0.981
High	82 (2.43)	3.11 (2.00, 4.83)	< 0.001	1.60 (1.02, 2.49)	0.039	1.38 (0.71, 2.67)	0.339
*P* for trend		< 0.001		0.039		0.319	
NFS							
Low	28 (0.64)	Reference		Reference		Reference	
Intermediate	92 (1.68)	3.47 (1.87, 6.43)	< 0.001	3.01 (1.63, 5.56)	< 0.001	2.15 (1.10, 4.21)	0.025
High	46 (4.44)	10.68 (5.12, 22.27)	< 0.001	6.75 (3.27, 13.90)	< 0.001	3.54 (1.65, 7.60)	0.001
*P* for trend		< 0.001		< 0.001		< 0.001	
FIB-4							
Low	106 (1.23)	Reference		Reference		Reference	
Intermediate	43 (1.79)	1.77 (1.13, 2.78)	0.013	1.56 (0.97, 2.53)	0.068	1.19 (0.74, 1.91)	0.480
High	17 (5.24)	5.40 (2.96, 9.85)	< 0.001	3.63 (1.92, 6.85)	< 0.001	2.25 (1.17, 4.33)	0.015
*P* for trend		< 0.001		< 0.001		0.035	
SAFE							
Low	28 (0.60)	Reference		Reference		Reference	
Intermediate	61 (1.66)	3.11 (1.68, 5.74)	< 0.001	2.57 (1.39, 4.76)	0.003	1.79 (0.90, 3.56)	0.094
High	77 (4.72)	10.06 (5.61, 18.05)	< 0.001	6.49 (3.56, 11.81)	< 0.001	3.63 (1.77, 7.44)	< 0.001
*P* for trend		< 0.001		< 0.001		< 0.001	
APRI							
Low	159 (1.39)	Reference		Reference		Reference	
High	7 (4.11)	3.40 (1.47, 7.90)	0.004	2.14 (0.89, 5.15)	0.090	1.95 (0.75, 5.02)	0.169

Model 1: Unadjusted

Model 2: Adjusted for age, sex, race/ethnicity, education level, family PIR, marital status, smoking, drinking, and physical activity

Model 3: Adjusted for Model 2 variables plus BMI, eGFR, hypertension, diabetes, hyperlipidemia, and NAFLD

For each fibrosis score, variables used in the calculation of that score were not additionally included in the corresponding multivariable model, as described in the Methods

*APRI* Aspartate aminotransferase to platelet ratio index score, *BMI* Body mass index, *CVD* Cardiovascular disease, *CI* Confidence interval, *CKM* Cardiovascular-kidney-metabolic, *eGFR* estimated glomerular filtration rate, *FIB-4* Fibrosis-4 index score, *LiverRisk* LiverRisk score, *MAF-5* metabolic dysfunction-associated fibrosis-5 score, *NAFLD* Nonalcoholic fatty liver disease, *NFS* Nonalcoholic fatty liver disease fibrosis score, *PIR* Poverty income ratio, *SAFE* Steatosis-associated fibrosis estimator score, *SD* Standard deviation, *sHR* Subdistribution hazard ratio

To further examine the dose-response relationships between the liver fibrosis scores and both all-cause and CVD mortality, we performed RCS analyses (Supplementary Figure S2). In the fully adjusted models, all six noninvasive liver fibrosis scores demonstrated significant overall associations with all-cause mortality (all *P* overall < 0.001). Regarding CVD mortality, significant overall associations were observed for the LiverRisk, NFS, FIB-4, and SAFE scores (all *P* overall < 0.05). In contrast, no significant dose-response associations were observed for MAF-5 or APRI.

### Subgroup and sensitivity analyses

To further assess the robustness of these findings, we conducted interaction and subgroup analyses (Supplementary Tables S4–S7). Across subgroups defined by age, sex, and race/ethnicity, the associations of the noninvasive liver fibrosis scores with mortality risks remained generally consistent. However, significant interactions were observed for the NFS and SAFE scores according to advanced CKM syndrome status (all *P* for interaction < 0.05). Specifically, the associations of the NFS and SAFE scores with all-cause mortality were substantially stronger among participants without advanced CKM syndrome (HR = 1.78 and 1.74, respectively) than in those with advanced CKM syndrome (HR = 1.41 and 1.49, respectively). Notably, regarding CVD mortality, the significant associations of NFS (sHR = 2.03) and SAFE (sHR = 1.68) observed in the non-advanced group were attenuated and no longer statistically significant in the advanced CKM group (sHR = 0.98 and 1.06, respectively). Across all three sensitivity analyses, the results were not materially altered. The LiverRisk, NFS, FIB-4, and SAFE scores consistently demonstrated significant associations with both mortality outcomes (Supplementary Tables S8–S13).

### Incremental predictive value of noninvasive liver fibrosis scores

**Fig. 3:**
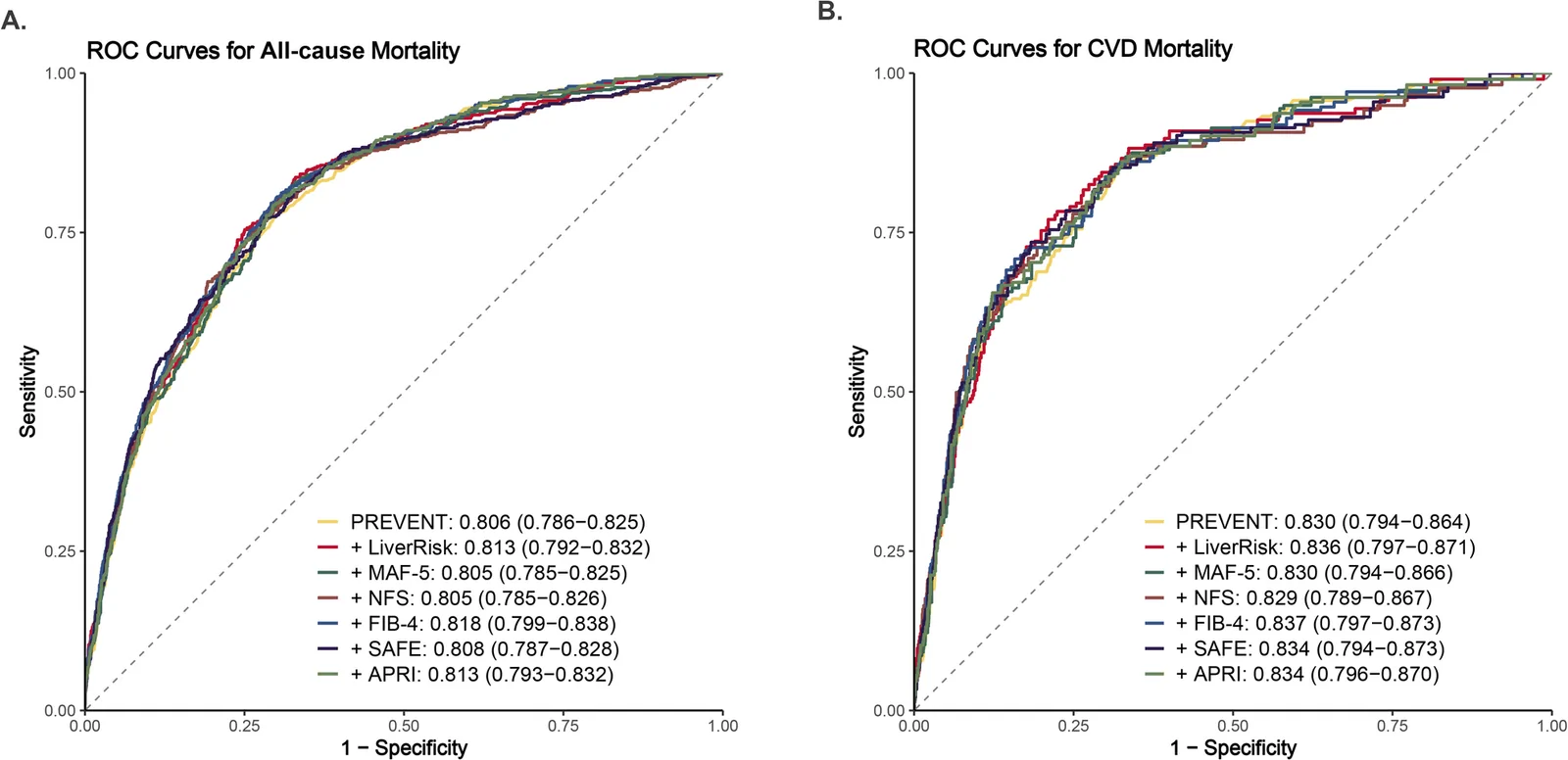
ROC curves for the predictive performance of noninvasive liver fibrosis scores. ROC curves for predicting all-cause **A** and CVD **B** mortality at 10-year follow-up. APRI, aspartate aminotransferase to platelet ratio index score; FIB-4, Fibrosis-4 score; LiverRisk, LiverRisk score; MAF-5, metabolic dysfunction-associated fibrosis 5 score; NFS, nonalcoholic fatty liver disease fibrosis score; SAFE, steatosis-associated fibrosis estimator score

The predictive performance of the base PREVENT model and the updated models incorporating fibrosis scores is presented in Fig. 3. The baseline PREVENT equations effectively predicted 10-year all-cause (AUC = 0.806) and CVD mortality (AUC = 0.830). Among the six noninvasive liver fibrosis scores, FIB-4 demonstrated the most pronounced incremental prognostic value, improving the AUCs to 0.818 for all-cause mortality and 0.837 for CVD mortality. The LiverRisk, APRI, and SAFE scores also offered slight improvements in discrimination, whereas the inclusion of MAF-5 and NFS did not.

## Discussion

In this nationally representative cohort of U.S. adults, we found that several noninvasive liver fibrosis scores were closely associated with both CKM syndrome severity and adverse long-term outcomes. Specifically, higher LiverRisk, MAF-5, NFS, FIB-4, and SAFE scores were associated with increased risk of advanced CKM syndrome, whereas APRI was not. In addition, all six fibrosis scores were significantly associated with a higher risk of all-cause mortality, while only LiverRisk, NFS, FIB-4, and SAFE showed robust associations with CVD mortality in competing-risk analyses. These associations were further supported by dose-response analyses and remained generally consistent across subgroup and sensitivity analyses. Finally, when added individually to the PREVENT equations, FIB-4 showed the greatest incremental prognostic value for both all-cause and CVD mortality, whereas LiverRisk, SAFE, and APRI provided only modest improvement.

There is substantial interorgan crosstalk among the cardiovascular system, liver, and kidneys. Dysfunction in any one of these organs may adversely influence the others through shared mechanisms, including chronic inflammation, oxidative stress, metabolic dysregulation, mitochondrial dysfunction, endothelial injury, and extracellular matrix remodeling [28–31]. In this context, liver fibrosis may reflect not only hepatic injury itself, but also broader systemic disturbances relevant to CKM syndrome. Moreover, liver fibrosis may also be linked to liver-specific pathways that influence cardiovascular risk. Experimental studies have shown that extracellular vesicles derived from steatotic hepatocytes can directly promote endothelial inflammation and atherogenesis, supporting a liver-to-vessel signaling pathway [32]. Taken together, these findings suggest that the association between liver fibrosis and adverse CKM outcomes may be driven not only by shared metabolic dysregulation, but also by partially independent liver-derived mechanisms. This concept is further supported by emerging therapeutic evidence. Incretin-based therapies, which target excess adiposity, insulin resistance, and systemic metabolic dysfunction, may provide benefits across the cardiovascular, liver, and kidney axes [33]. In a prespecified analysis of the SELECT trial, semaglutide reduced major adverse cardiovascular events in patients at high risk of substantial liver fibrosis and also improved fatty liver-related indices [34].

Noninvasive liver fibrosis scores have value beyond the assessment of liver fibrosis itself, as they may also provide clinically meaningful information for broader risk stratification [15, 35]. More recently, several studies have reported associations between liver fibrosis and CKM syndrome. Hu et al. reported that liver fibrosis assessed by transient elastography was significantly associated with advanced CKM syndrome [17]. Liu et al., using NHANES III data, further showed that NAFLD was highly prevalent in individuals with CKM syndrome and was associated with an increased risk of all-cause mortality [18]; Chen et al. found that CKM stages 3–4 were an independent risk factor for liver fibrosis in patients with metabolic dysfunction-associated steatotic liver disease (MASLD), highlighting the substantial overlap between these two conditions [36]; Similarly, Zhou et al. reported that higher CKM stages were associated not only with more severe liver fibrosis, but also with fibrosis progression and an increased risk of liver-related events in MASLD [37]. Ren et al. found that liver fibrosis assessed by the NFS was associated with CKM progression in patients with type 2 diabetes mellitus [38]. Recently, Cao et al. also reported that higher FIB-4 and NFS were associated with increased risks of both all-cause mortality and cardiovascular mortality [39]. Compared with previous studies focusing on individual fibrosis markers, our study provided a more comprehensive comparison of multiple commonly used noninvasive liver fibrosis scores within the CKM framework. Consistent with these previous findings, our study further extended the literature by evaluating a broader range of noninvasive liver fibrosis scores. In addition to FIB-4 and NFS, we also assessed LiverRisk, MAF-5, SAFE, and APRI, and found that all six scores were significantly associated with all-cause mortality in the context of CKM syndrome. For CVD mortality, significant associations were observed for LiverRisk, SAFE, NFS, and FIB-4. These findings remained unchanged in sensitivity analyses, supporting the robustness of our results. Notably, subgroup analyses revealed significant interactions between CKM status and the associations of NFS and SAFE with CVD mortality. Specifically, both NFS and SAFE were significantly associated with CVD mortality in participants without advanced CKM syndrome, whereas these associations were attenuated in those with advanced CKM syndrome. Although no significant interaction was observed for LiverRisk or FIB-4, their associations with CVD mortality also appeared to be stronger in the non-advanced CKM subgroup. One possible explanation is that, in individuals without advanced CKM syndrome, liver fibrosis scores may reflect early metabolic and vascular abnormalities not fully captured by CKM staging, resulting in stronger associations with subsequent CVD mortality. In contrast, among those with advanced CKM syndrome, the already high cardiovascular risk may make these associations less evident. The significant interactions observed for NFS and SAFE may further suggest that these scores are more sensitive to early multisystem risk. These exploratory subgroup findings also suggest that, among individuals without advanced CKM syndrome, noninvasive liver fibrosis assessment may have greater value for early risk identification and stratification. Early identification in this subgroup may be clinically relevant, because timely lifestyle interventions—particularly sustained weight loss through dietary modification and increased physical activity—are recommended as the cornerstone of management for steatotic liver disease and may help attenuate fibrosis progression [40].

### Limitations

Several limitations of this study should be acknowledged. First, this was a retrospective cohort study, which precludes causal inference regarding the associations of noninvasive liver fibrosis scores with CKM severity and clinical outcomes. Second, noninvasive liver fibrosis scores were calculated only at baseline, and longitudinal changes over time could not be assessed. Therefore, further studies are needed to determine whether temporal changes in these scores are associated with subsequent mortality risk. Third, liver fibrosis was assessed using noninvasive fibrosis scores rather than histology or transient elastography. Although these scores are practical and widely used in large-scale population studies, they remain indirect measures of liver fibrosis and may have resulted in misclassification. Fourth, although we adjusted for multiple potential confounders, residual confounding from unmeasured factors, such as genetic susceptibility and other unrecorded clinical variables, cannot be excluded. And the use of clinically defined categorical covariates instead of continuous metabolic measures may have resulted in some loss of information and residual confounding. Fifth, although we explored whether adding noninvasive liver fibrosis scores to the PREVENT model could improve predictive performance and observed modest improvements in AUC, the clinical significance of these improvements remains uncertain. Further studies are needed to evaluate the prognostic utility of incorporating noninvasive liver fibrosis scores into routine clinical risk assessment and to determine whether liver fibrosis scores better tailored to the CKM framework could further improve risk stratification. Finally, because the PREVENT equations are not applicable to individuals aged < 30 years or ≥ 80 years, these participants were excluded from the present analysis. This restriction may have introduced selection bias and may limit the generalizability of our findings to younger and older populations. Therefore, our findings should be interpreted with caution and further validated in prospective studies.

## Conclusion

In conclusion, this nationwide cohort study showed that multiple noninvasive liver fibrosis scores were significantly associated with CKM severity and adverse clinical outcomes. Higher LiverRisk, MAF-5, NFS, FIB-4, and SAFE scores were associated with advanced CKM syndrome, whereas all six scores were associated with all-cause mortality. LiverRisk, NFS, FIB-4, and SAFE showed more consistent associations with CVD mortality.

## Supplementary Information


Supplementary Material 1.


## Data Availability

This study analyzed data obtained from the publicly available NHANES database. For more comprehensive information on the database, including its design, data collection procedures, and methodology, readers are referred to consult the official NHANES website: https://www.cdc.gov/nchs/nhanes/.

## Abbreviations

ALB: Albumin
ALT: Alanine aminotransferase
APRI: Aspartate aminotransferase to platelet ratio index
AST: Aspartate aminotransferase
AUC: Area under the curve
BMI: Body mass index
BUN: Blood urea nitrogen
CI: Confidence intervals
CKM: Cardiovascular-kidney-metabolic
CVD: Cardiovascular disease
DBP: Diastolic blood pressure
eGFR: Estimated glomerular filtration rate
FIB-4: Fibrosis-4 score
FPG: Fasting blood glucose
GGT: Gamma-glutamyl transferase
GLB: Globulin
HBA1C: Haemoglobin A1c
HDL-c: High-density lipoprotein cholesterol
HR: Hazard ratio
LDL-c: Low-density lipoprotein cholesterol
LiverRisk: Liverrisk score
MAF-5: Metabolic dysfunction-associated fibrosis 5
MASLD: Metabolic dysfunction-associated steatotic liver disease
NAFLD: Nonalcoholic fatty liver disease
NDI: Nationality death index
NFS: Nonalcoholic fatty liver disease fibrosis score
NHANES: National health and nutrition examination survey
OR: Odds ratio
PIR: Poverty income ratio
PLT: Platelets
RCS: Restricted cubic spline
SAFE: Steatosis-associated fibrosis estimator
SBP: Systolic blood pressure
Scr: Serum creatinine
SD: Standard deviation
TC: Total cholesterol
TG: Triglyceride
UA: Uric acid
UACR: Urine albumin-to-creatinine ratio
VIF: Variance inflation factor
WC: Waist circumference
