# Supplementary material for: Association of noninvasive liver fibrosis scores with severity and outcomes among individuals with cardiovascular-kidney-metabolic syndrome: evidence from the NHANES 2005–2018
Source: BMC Cardiovasc Disord. 2026 Jun 22;26:765. doi: 10.1186/s12872-026-06148-2 (PMC13536672; doi:10.1186/s12872-026-06148-2)
Supplement: Supplementary file 1 [file 12872_2026_6148_MOESM1_ESM.docx]

| **Contents** | **Pages** |
| --- | --- |
| **Supplementary method:** |  |
| Assessment of CKM syndrome stages | 2 |
| Noninvasive liver fibrosis scores | 3 |
| **Figures** |  |
| Figure S1. Flowchart of the study | 4 |
| Figure S2. Restricted cubic spline analyses of liver fibrosis and mortality outcomes | 5 |
| **Tables** |  |
| Table S1. Summary of excluded covariates to prevent over-adjustment for each liver fibrosis score | 6 |
| Table S2. Comparison of noninvasive liver fibrosis scores between non-advanced and advanced CKM syndrome | 7 |
| Table S3. Comparison of baseline characteristics between analyzed cohort and excluded cohort | 8 |
| Table S4. Stratified analyses of the LiverRisk score and mortality risks among participants with CKM syndrome (fully adjusted model) | 11 |
| Table S5. Stratified analyses of the NFS score and mortality risks among participants with CKM syndrome (fully adjusted model) | 12 |
| Table S6. Stratified analyses of the FIB-4 score and mortality risks among participants with CKM syndrome (fully adjusted model) | 13 |
| Table S7. Stratified analyses of the SAFE score and mortality risks among participants with CKM syndrome (fully adjusted model) | 14 |
| Table S8. Associations between noninvasive liver fibrosis scores and all-cause mortality among participants with CKM syndrome (after multiple imputation) | 15 |
| Table S9. Associations between noninvasive liver fibrosis scores and CVD mortality among participants with CKM syndrome (after multiple imputation) | 18 |
| Table S10. Associations between noninvasive liver fibrosis scores and all-cause mortality among participants with CKM syndrome (excluding accidental mortality) | 20 |
| Table S11. Associations between noninvasive liver fibrosis scores and CVD mortality among participants with CKM syndrome (excluding accidental mortality) | 22 |
| Table S12. Associations between noninvasive liver fibrosis scores and all-cause mortality among participants with CKM syndrome (excluding events within two years) | 24 |
| Table S13. Associations between noninvasive liver fibrosis scores and CVD mortality among participants with CKM syndrome (excluding events within two years) | 26 |

**Supplementary method:**

**Assessment of CKM syndrome stages**

CKM Stage 0 was defined as having both a normal body mass index (BMI) and a normal waist circumference, without meeting the criteria for any other stage. The specific criteria were as follows: BMI: <23 kg/m² for individuals of Asian ethnicity; <25 kg/m² for all other racial and ethnic groups. Waist Circumference: Asian: <80 cm for women; <90 cm for men; All other groups: <88 cm for women; <102 cm for men.

CKM Stage 1 was assigned to individuals who met one or more of the following criteria: (1) elevated BMI: ≥23 kg/m² for individuals of Asian race; ≥25 kg/m² for all other racial and ethnic groups; (2) elevated waist circumference: Asian race: ≥80 cm for women; ≥90 cm for men; other races/ethnicities: ≥88 cm for women; ≥102 cm for men; (3) prediabetes, defined as glycated hemoglobin (HbA1c) of 5.7% to <6.5%, or fasting blood glucose of 100 mg/dL to <126 mg/dL.

CKM Stage 2 was defined by the presence of either metabolic risk factors or moderate- to high-risk chronic kidney disease (CKD), as per the American Heart Association (AHA) recommendations which adopt the Kidney Disease Improving Global Outcomes (KDIGO) criteria. Qualifying metabolic risk factors included: (1) elevated fasting triglycerides (≥135 mg/dL), (2) hypertension, (3) diabetes, or (4) metabolic syndrome. Metabolic syndrome was defined as ≥3 of the following components: (1) elevated waist circumference (using stage 1 criteria), (2) Low HDL-C: <40 mg/dL in men or <50 mg/dL in women, (3) elevated fasting triglycerides (≥150 mg/dL), (4) elevated blood pressure (SBP ≥130 or DBP ≥80 mmHg, or use of antihypertensive medication), (5) prediabetes (as defined in stage 1). CKD stage was determined based on estimates of estimated glomerular filtration rate (eGFR) and urinary albumin-to-creatinine ratio (UACR).

CKM Stage 3 was defined by the presence of either very-high-risk CKD as classified by KDIGO criteria, or a high predicted 10-year cardiovascular disease (CVD) risk (≥20%). The 10-year CVD risk was estimated using the AHA Predicting Risk of CVD EVENTs (PREVENT) equations (https://professional.heart.org/en/guidelinesand-statements/prevent-calculator). This ≥20% risk threshold aligns with the recommended cut-point for high risk provided by the AHA.

CKM Stage 4 was identified based on self-reported established cardiovascular disease (coronary heart disease, angina, heart attack, heart failure, and stroke).

**Noninvasive liver fibrosis scores**

Six noninvasive liver fibrosis scores were used to assess the liver fibrosis, including LiverRisk, MAF-5, NFS, FIB-4, SAFE, and APRI score. Except for LiverRisk score, which was calculated via the online website (https://www.liverriskscore.com), the other 5 formulas are as follows:

$$MAF-5=-11.3674+WC*0.0282-BMI*0.1761+WC*BMI*0.0019+2.0762*diabetes \left( yes=1,no=0 \right)+\ln\left( \mathrm{AST} \right)*2.9207-platelets*0.0059$$

$$NFS=-1.675+0.037*Age+0.094*BMI+1.13*(pre)diabetes \left( yes=1,no=0 \right)+0.99*AST/ALT-0.013*platelets-0.66*albumin$$

$$FIB-4=Age*AST/(platelets*\mathrm{ALT}^{0.5})$$

$$SAFE=2.97*Age+5.99*BMI+154.85*ln\left( \mathrm{AST} \right)-58.23*ln\left( \mathrm{ALT} \right)+195.48*\ln\left( \mathrm{globulin} \right)-141.61*\ln\left( \mathrm{platelets} \right)+62.85*diabetes \left( yes=1,no=0 \right)-75$$

$$APRI=[(AST/upper limit of normal)/platelets] * 100$$

**Figure S1. Flowchart of the study**

**Figure S2. Restricted cubic spline analyses of noninvasive liver fibrosis and mortality outcomes**

Restricted cubic spline models illustrate the association between noninvasive liver fibrosis and all-cause mortality for LiverRisk (**A**), MAF-5 (**B**), NFS (**C**), FIB-4 (**D**), SAFE (**E**), and APRI (**F**). Restricted cubic spline models illustrate the association between noninvasive liver fibrosis and CVD mortality for the LiverRisk (**G**), MAF-5 (**H**), NFS (**I**), FIB-4 (**J**), SAFE (**K**), and APRI (**L**). Solid red lines represent adjusted HRs, with shaded areas indicating 95% CIs. APRI, aspartate aminotransferase to platelet ratio index score; CI, confidence intervals; FIB-4, Fibrosis-4 score; HRs, hazard ratios; LiverRisk, LiverRisk score; MAF-5, metabolic dysfunction-associated fibrosis-5 score; NFS, nonalcoholic fatty liver disease fibrosis score; and SAFE, steatosis-associated fibrosis estimator score.

**Table S1. Summary of excluded covariates to prevent over-adjustment for each liver fibrosis score**

| **Noninvasive liver fibrosis score** | **Excluded variables** |
| --- | --- |
| LiverRisk | Age, sex |
| MAF-5 | BMI, diabetes |
| NFS | Age, BMI, diabetes |
| FIB-4 | Age |
| SAFE | Age, BMI, diabetes |
| APRI | None |

APRI, aspartate aminotransferase to platelet ratio index score; CKM, cardiovascular-kidney-metabolic; FIB-4, Fibrosis-4 score; LiverRisk, LiverRisk score; MAF-5, metabolic dysfunction-associated fibrosis-5 score; NFS, nonalcoholic fatty liver disease fibrosis score; PIR, poverty income ratio; and SAFE, steatosis-associated fibrosis estimator score.

**Table S2. Comparison of noninvasive liver fibrosis scores between non-advanced and advanced CKM syndrome**

| **Liver fibrosis score** | **Overall n = 8592 (100.00%)** | **Non-advanced CKM n = 7205 (87.69%)** | **Advanced CKM n = 1387 (12.31%)** | ***P*** |
| --- | --- | --- | --- | --- |
| Continuous variables |  |  |  |  |
| LiverRisk | 5.45 ± 0.02 | 5.32 ± 0.02 | 6.37 ± 0.05 | <0.001 |
| MAF-5 | 0.17 ± 0.04 | -0.02 ± 0.04 | 1.56 ± 0.09 | <0.001 |
| NFS | -1.30 ± 0.02 | -1.47 ± 0.02 | -0.08 ± 0.04 | <0.001 |
| FIB-4 | 1.16 ± 0.01 | 1.09 ± 0.01 | 1.66 ± 0.03 | <0.001 |
| SAFE | -5.33 ± 1.49 | -17.56 ± 1.49 | 81.82 ± 2.94 | <0.001 |
| APRI | 0.35 ± 0.01 | 0.34 ± 0.01 | 0.38 ± 0.01 | <0.001 |
| Categorical variables |  |  |  |  |
| LiverRisk |  |  |  | <0.001 |
| Low | 6,367 (78.27%) | 5,744 (82.72%) | 623 (46.58%) |  |
| Intermediate | 2,050 (20.31%) | 1,346 (16.13%) | 704 (50.05%) |  |
| High | 175 (1.42%) | 115 (1.15%) | 60 (3.37%) |  |
| MAF-5 |  |  |  | <0.001 |
| Low | 4,490 (55.21%) | 4,116 (58.89%) | 374 (28.99%) |  |
| Intermediate | 1,393 (16.23%) | 1,167 (16.19%) | 226 (16.51%) |  |
| High | 2,709 (28.56%) | 1,922 (24.92%) | 787 (54.50%) |  |
| NFS |  |  |  | <0.001 |
| Low | 3,644 (46.26%) | 3,455 (50.63%) | 189 (15.11%) |  |
| Intermediate | 4,038 (45.50%) | 3,267 (43.93%) | 771 (56.71%) |  |
| High | 910 (8.24%) | 483 (5.44%) | 427 (28.18%) |  |
| FIB-4 |  |  |  | <0.001 |
| Low | 6,635 (78.88%) | 5,727 (80.69%) | 908 (65.94%) |  |
| Intermediate | 1,693 (18.78%) | 1,341 (17.76%) | 352 (26.06%) |  |
| High | 264 (2.34%) | 137 (1.54%) | 127 (8.01%) |  |
| SAFE |  |  |  | <0.001 |
| Low | 4,152 (56.19%) | 3,970 (61.75%) | 182 (16.57%) |  |
| Intermediate | 2,998 (32.05%) | 2,428 (30.54%) | 570 (42.83%) |  |
| High | 1,442 (11.75%) | 807 (7.71%) | 635 (40.60%) |  |
| APRI |  |  |  | 0.094 |
| Low | 8,436 (98.50%) | 7,087 (98.58%) | 1,349 (97.95%) |  |
| High | 156 (1.50%) | 118 (1.42%) | 38 (2.05%) |  |

APRI, aspartate aminotransferase to platelet ratio index score; CKM, cardiovascular-kidney-metabolic; FIB-4, Fibrosis-4 score; LiverRisk, LiverRisk score; MAF-5, metabolic dysfunction-associated fibrosis-5 score; NFS, nonalcoholic fatty liver disease fibrosis score; PIR, poverty income ratio; and SAFE, steatosis-associated fibrosis estimator score.

**Table S3. Comparison of baseline characteristics between analyzed cohort and excluded cohort**

| **Characteristic** | **Overall,  n = 12443 (100.00%)** | **Analyzed cohort,  n = 8592 (69.05%)** | **Excluded cohort,  n = 3851 (30.95%)** | ***P*** |
| --- | --- | --- | --- | --- |
| Age | 52.85 ± 13.53 | 52.32 ± 13.41 | 54.04 ± 13.73 | <0.001 |
| Sex |  |  |  | >0.900 |
| Female | 6,392 (51.37%) | 4,410 (51.33%) | 1,982 (51.47%) |  |
| Male | 6,051 (48.63%) | 4,182 (48.67%) | 1,869 (48.53%) |  |
| Race/ethnicity |  |  |  | <0.001 |
| Mexican American | 1,972 (15.85%) | 1,316 (15.32%) | 656 (17.03%) |  |
| Non-Hispanic Black | 2,592 (20.83%) | 1,720 (20.02%) | 872 (22.64%) |  |
| Non-Hispanic White | 5,117 (41.12%) | 3,781 (44.01%) | 1,336 (34.69%) |  |
| Other | 2,762 (22.20%) | 1,775 (20.66%) | 987 (25.63%) |  |
| Education level |  |  |  | <0.001 |
| Under high school | 3,198 (25.72%) | 1,892 (22.02%) | 1,306 (34.00%) |  |
| High school or equivalent | 2,787 (22.42%) | 1,936 (22.53%) | 851 (22.16%) |  |
| Above high school | 6,448 (51.86%) | 4,764 (55.45%) | 1,684 (43.84%) |  |
| Family PIR |  |  |  | <0.001 |
| <3 | 6,896 (60.70%) | 4,964 (57.77%) | 1,932 (69.77%) |  |
| >=3 | 4,465 (39.30%) | 3,628 (42.23%) | 837 (30.23%) |  |
| Marital status |  |  |  | 0.200 |
| Married or living with a partner | 7,039 (56.57%) | 4,827 (56.18%) | 2,212 (57.44%) |  |
| Other | 5,404 (43.43%) | 3,765 (43.82%) | 1,639 (42.56%) |  |
| Smoking status |  |  |  | <0.001 |
| Never smoker | 6,654 (53.52%) | 4,718 (54.91%) | 1,936 (50.40%) |  |
| Former smoker | 3,247 (26.12%) | 2,177 (25.34%) | 1,070 (27.86%) |  |
| Current smoker | 2,532 (20.37%) | 1,697 (19.75%) | 835 (21.74%) |  |
| Drinking status |  |  |  | <0.001 |
| Nondrinker | 3,350 (32.42%) | 2,693 (31.34%) | 657 (37.72%) |  |
| Low-to-moderate drinker | 5,994 (58.00%) | 5,066 (58.96%) | 928 (53.27%) |  |
| Heavy drinker | 990 (9.58%) | 833 (9.70%) | 157 (9.01%) |  |
| Physical activity |  |  |  | <0.001 |
| Less than moderate | 5,433 (43.66%) | 3,536 (41.15%) | 1,897 (49.26%) |  |
| Moderate | 3,740 (30.06%) | 2,708 (31.52%) | 1,032 (26.80%) |  |
| Vigorous | 3,270 (26.28%) | 2,348 (27.33%) | 922 (23.94%) |  |
| BMI | 29.72 ± 7.04 | 29.55 ± 6.76 | 30.11 ± 7.64 | <0.001 |
| SBP | 124.81 ± 18.41 | 124.50 ± 17.91 | 125.59 ± 19.59 | 0.045 |
| DBP | 71.28 ± 11.44 | 71.37 ± 11.38 | 71.07 ± 11.59 | 0.120 |
| Platelets | 244.39 ± 66.90 | 244.60 ± 66.49 | 243.92 ± 67.82 | 0.600 |
| BUN | 13.61 ± 5.65 | 13.50 ± 5.34 | 13.88 ± 6.32 | 0.400 |
| Scr | 0.89 ± 0.47 | 0.88 ± 0.34 | 0.92 ± 0.70 | 0.008 |
| UA | 5.53 ± 1.44 | 5.52 ± 1.42 | 5.54 ± 1.50 | 0.900 |
| ALT | 25.56 ± 19.42 | 25.61 ± 18.20 | 25.43 ± 22.01 | 0.072 |
| AST | 25.70 ± 21.42 | 25.72 ± 21.31 | 25.66 ± 21.68 | 0.500 |
| GGT | 31.61 ± 43.50 | 30.85 ± 41.40 | 33.38 ± 48.00 | 0.009 |
| HbA1c | 5.87 ± 1.18 | 5.81 ± 1.11 | 6.01 ± 1.30 | <0.001 |
| FBG | 112.17 ± 38.30 | 110.66 ± 35.46 | 115.53 ± 43.79 | <0.001 |
| TG | 131.42 ± 117.04 | 120.80 ± 66.82 | 155.95 ± 184.84 | <0.001 |
| LDL-c | 116.59 ± 35.71 | 116.97 ± 35.27 | 115.67 ± 36.76 | 0.024 |
| HDL-c | 53.82 ± 16.20 | 54.63 ± 16.13 | 51.98 ± 16.21 | <0.001 |
| TC | 196.01 ± 41.77 | 195.75 ± 39.99 | 196.60 ± 45.62 | 0.700 |
| UACR | 50.68 ± 352.36 | 42.14 ± 304.99 | 70.32 ± 441.94 | <0.001 |
| eGFR | 91.93 ± 20.81 | 92.06 ± 20.12 | 91.64 ± 22.34 | 0.400 |
| Diabetes |  |  |  | <0.001 |
| No | 9,691 (77.88%) | 6,874 (80.00%) | 2,817 (73.15%) |  |
| Yes | 2,752 (22.12%) | 1,718 (20.00%) | 1,034 (26.85%) |  |
| Hypertension |  |  |  | 0.014 |
| No | 5,268 (42.34%) | 3,701 (43.07%) | 1,567 (40.69%) |  |
| Yes | 7,175 (57.66%) | 4,891 (56.93%) | 2,284 (59.31%) |  |
| NAFLD |  |  |  | <0.001 |
| No | 7,714 (69.65%) | 5,744 (66.85%) | 1,970 (79.31%) |  |
| Yes | 3,362 (30.35%) | 2,848 (33.15%) | 514 (20.69%) |  |
| LiverRisk | 5.66 ± 1.63 | 5.60 ± 1.55 | 5.79 ± 1.78 | <0.001 |
| MAF-5 | 0.36 ± 2.32 | 0.31 ± 2.29 | 0.51 ± 2.39 | <0.001 |
| NFS | -1.10 ± 1.49 | -1.15 ± 1.48 | -0.97 ± 1.53 | <0.001 |
| FIB-4 | 1.24 ± 0.84 | 1.22 ± 0.80 | 1.28 ± 0.91 | <0.001 |
| SAFE | 15.71 ± 96.92 | 11.28 ± 95.32 | 26.62 ± 99.94 | <0.001 |
| APRI | 0.35 ± 0.38 | 0.35 ± 0.41 | 0.35 ± 0.33 | 0.600 |

Continuous variable expressed as mean ± SD; Categorical variable expressed as n (%).

ALT, alanine aminotransferase; APRI, aspartate aminotransferase to platelet ratio index score; AST, aspartate aminotransferase; BMI, body mass index; BUN, blood urea nitrogen; CKM, cardiovascular-kidney-metabolic; DBP, diastolic blood pressure; eGFR, estimated glomerular filtration rate; FBG, fasting blood glucose; FIB-4, Fibrosis-4 score; GGT, gamma-glutamyl transferase; HbA1c, haemoglobin A1c; HDL-c, high-density lipoprotein cholesterol; LDL-c, low-density lipoprotein cholesterol; LiverRisk, LiverRisk score; MAF-5, metabolic dysfunction-associated fibrosis-5 score; NAFLD, nonalcoholic fatty liver disease; NFS, nonalcoholic fatty liver disease fibrosis score; PIR, poverty income ratio; SAFE, steatosis-associated fibrosis estimator score; SBP, systolic blood pressure; SD, standard deviation; Scr, serum creatinine; TC, total cholesterol; TG, triglyceride; UA, uric acid; and UACR, urine albumin-to-creatinine ratio.

**Table S4.** **Stratified analyses of the LiverRisk score and mortality risks among participants with CKM syndrome (fully adjusted model)**

| **Subgroup** | **All-cause mortality** | | |  | **CVD mortality** | | |
| --- | --- | --- | --- | --- | --- | --- | --- |
|  | ***HR* (95% *CI*)** | ***P*** | ***P* for interaction** |  | ***sHR* (95% *CI*)** | ***P*** | ***P* for interaction** |
| Age |  |  | 0.137 |  |  |  | 0.400 |
| < 60 | 1.20 (1.12, 1.28) | <0.001 |  |  | 1.11 (1.02, 1.22) | 0.018 |  |
| >= 60 | 1.38 (1.27, 1.50) | <0.001 |  |  | 1.33 (0.99, 1.78) | 0.057 |  |
| Sex |  |  | 0.049 |  |  |  | 0.524 |
| Female | 1.34 (1.22, 1.46) | <0.001 |  |  | 1.03 (0.81, 1.30) | 0.809 |  |
| Male | 1.21 (1.15, 1.28) | <0.001 |  |  | 1.13 (1.00, 1.27) | 0.047 |  |
| Race/ethnicity |  |  | 0.392 |  |  |  | 0.990 |
| Mexican American | 1.17 (0.90, 1.53) | 0.242 |  |  | 0.97 (0.54, 1.75) | 0.928 |  |
| Non-Hispanic Black | 1.28 (1.20, 1.36) | <0.001 |  |  | 1.14 (1.01, 1.30) | 0.042 |  |
| Non-Hispanic White | 1.24 (1.15, 1.34) | <0.001 |  |  | 1.18 (1.03, 1.34) | 0.014 |  |
| Other | 1.49 (1.23, 1.80) | <0.001 |  |  | 1.12 (0.82, 1.55) | 0.474 |  |
| Advanced CKM |  |  | 0.858 |  |  |  | 0.397 |
| No | 1.22 (1.13, 1.31) | <0.001 |  |  | 1.13 (1.03, 1.24) | 0.011 |  |
| Yes | 1.27 (1.18, 1.37) | <0.001 |  |  | 1.11 (0.94, 1.31) | 0.209 |  |

CI, confidence interval; CKM, cardiovascular-kidney-metabolic; CVD, cardiovascular disease; HR, hazard ratio; sHR, subdistribution hazard ratio.

**Table S5.** **Stratified analyses of the NFS score and mortality risks among participants with CKM syndrome (fully adjusted model)**

| **Subgroup** | **All-cause mortality** | | |  | **CVD mortality** | | |
| --- | --- | --- | --- | --- | --- | --- | --- |
|  | ***HR* (95% *CI*)** | ***P*** | ***P* for interaction** |  | ***sHR* (95% *CI*)** | ***P*** | ***P* for interaction** |
| Age |  |  | 0.864 |  |  |  | 0.283 |
| < 60 | 1.54 (1.18, 2.01) | 0.001 |  |  | 1.13 (0.70, 1.81) | 0.624 |  |
| >= 60 | 1.51 (1.28, 1.78) | <0.001 |  |  | 1.48 (1.16, 1.90) | 0.002 |  |
| Sex |  |  | 0.387 |  |  |  | 0.525 |
| Female | 1.73 (1.39, 2.16) | <0.001 |  |  | 1.44 (0.98, 2.13) | 0.066 |  |
| Male | 1.86 (1.51, 2.29) | <0.001 |  |  | 1.61 (1.19, 2.17) | 0.002 |  |
| Race/ethnicity |  |  | 0.579 |  |  |  | 0.016 |
| Mexican American | 1.57 (0.99, 2.49) | 0.055 |  |  | 0.87 (0.49, 1.56) | 0.644 |  |
| Non-Hispanic Black | 1.55 (1.25, 1.92) | <0.001 |  |  | 1.48 (1.01, 2.16) | 0.042 |  |
| Non-Hispanic White | 1.91 (1.60, 2.28) | <0.001 |  |  | 1.82 (1.35, 2.45) | <0.001 |  |
| Other | 1.57 (0.92, 2.67) | 0.095 |  |  | 0.61 (0.27, 1.39) | 0.237 |  |
| Advanced CKM |  |  | 0.016 |  |  |  | 0.018 |
| No | 1.78 (1.45, 2.20) | <0.001 |  |  | 2.03 (1.29, 3.21) | 0.002 |  |
| Yes | 1.41 (1.20, 1.66) | <0.001 |  |  | 0.98 (0.71, 1.34) | 0.896 |  |

CI, confidence interval; CKM, cardiovascular-kidney-metabolic; CVD, cardiovascular disease; HR, hazard ratio; NFS, nonalcoholic fatty liver disease fibrosis scores; sHR, subdistribution hazard ratio.

**Table S6.** **Stratified analyses of the FIB-4 score and mortality risks among participants with CKM syndrome (fully adjusted model)**

| **Subgroup** | **All-cause mortality** | | |  | **CVD mortality** | | |
| --- | --- | --- | --- | --- | --- | --- | --- |
|  | ***HR* (95% *CI*)** | ***P*** | ***P* for interaction** |  | ***sHR* (95% *CI*)** | ***P*** | ***P* for interaction** |
| Age |  |  | 0.052 |  |  |  | 0.146 |
| < 60 | 1.18 (1.12, 1.24) | <0.001 |  |  | 1.03 (0.82, 1.29) | 0.816 |  |
| >= 60 | 1.31 (1.22, 1.40) | <0.001 |  |  | 1.21 (1.04, 1.41) | 0.013 |  |
| Sex |  |  | 0.151 |  |  |  | 0.616 |
| Female | 1.31 (1.22, 1.41) | <0.001 |  |  | 1.10 (0.95, 1.26) | 0.195 |  |
| Male | 1.20 (1.13, 1.27) | <0.001 |  |  | 1.11 (0.99, 1.25) | 0.068 |  |
| Race/ethnicity |  |  | 0.116 |  |  |  | 0.689 |
| Mexican American | 1.28 (1.16, 1.41) | <0.001 |  |  | 1.13 (0.75, 1.70) | 0.560 |  |
| Non-Hispanic Black | 1.19 (1.11, 1.27) | <0.001 |  |  | 1.08 (0.95, 1.23) | 0.237 |  |
| Non-Hispanic White | 1.24 (1.16, 1.33) | <0.001 |  |  | 1.16 (1.03, 1.30) | 0.016 |  |
| Other | 1.53 (1.27, 1.84) | <0.001 |  |  | 0.66 (0.20, 2.15) | 0.492 |  |
| Advanced CKM |  |  | 0.737 |  |  |  | 0.479 |
| No | 1.23 (1.16, 1.30) | <0.001 |  |  | 1.18 (1.05, 1.32) | 0.006 |  |
| Yes | 1.24 (1.17, 1.32) | <0.001 |  |  | 1.08 (0.89, 1.30) | 0.440 |  |

CI, confidence interval; CKM, cardiovascular-kidney-metabolic; CVD, cardiovascular disease; FIB-4, Fibrosis-4; HR, hazard ratio; sHR, subdistribution hazard ratio.

**Table S7.** **Stratified analyses of the SAFE score and mortality risks among participants with CKM syndrome (fully adjusted model)**

| **Subgroup** | **All-cause mortality** | | |  | **CVD mortality** | | |
| --- | --- | --- | --- | --- | --- | --- | --- |
|  | ***HR* (95% *CI*)** | ***P*** | ***P* for interaction** |  | ***sH*R (95% *CI*)** | ***P*** | ***P* for interaction** |
| Age |  |  | 0.947 |  |  |  | 0.211 |
| < 60 | 1.57 (1.31, 1.88) | <0.001 |  |  | 1.11 (0.83, 1.49) | 0.478 |  |
| >= 60 | 1.67 (1.47, 1.90) | <0.001 |  |  | 1.52 (1.19, 1.94) | <0.001 |  |
| Sex |  |  | 0.893 |  |  |  | 0.354 |
| Female | 1.88 (1.64, 2.16) | <0.001 |  |  | 1.29 (0.94, 1.77) | 0.119 |  |
| Male | 1.71 (1.47, 1.99) | <0.001 |  |  | 1.52 (1.19, 1.93) | <0.001 |  |
| Race/ethnicity |  |  | 0.056 |  |  |  | 0.509 |
| Mexican American | 1.62 (1.33, 1.97) | <0.001 |  |  | 1.41 (0.88, 2.25) | 0.15 |  |
| Non-Hispanic Black | 1.53 (1.37, 1.72) | <0.001 |  |  | 1.34 (1.02, 1.77) | 0.034 |  |
| Non-Hispanic White | 1.84 (1.58, 2.14) | <0.001 |  |  | 1.60 (1.25, 2.05) | <0.001 |  |
| Other | 2.24 (1.50, 3.32) | <0.001 |  |  | 0.96 (0.40, 2.31) | 0.931 |  |
| Advanced CKM |  |  | 0.020 |  |  |  | 0.028 |
| No | 1.74 (1.49, 2.03) | <0.001 |  |  | 1.68 (1.28, 2.21) | <0.001 |  |
| Yes | 1.49 (1.29, 1.71) | <0.001 |  |  | 1.06 (0.72, 1.55) | 0.775 |  |

CI, confidence interval; CKM, cardiovascular-kidney-metabolic; CVD, cardiovascular disease; HR, hazard ratio; SAFE, steatosis-associated fibrosis estimator; sHR, subdistribution hazard ratio.

**Table S8. Associations between noninvasive liver fibrosis scores and all-cause mortality among participants with CKM syndrome (after multiple imputation)**

| **Noninvasive liver fibrosis score** | **Event, n (%)** | **Model 1** | |  | **Model 2** | |  | **Model 3** | |
| --- | --- | --- | --- | --- | --- | --- | --- | --- | --- |
|  |  | ***HR* (95% *CI*)** | ***P*** |  | ***HR* (95% *CI*)** | ***P*** |  | ***HR* (95% *CI*)** | ***P*** |
| Continuous variables (per 1 SD increase) |  |  |  |  |  |  |  |  |  |
| LiverRisk | 1092 (6.82) | 1.33 (1.26, 1.41) | <0.001 |  | 1.30 (1.23, 1.38) | <0.001 |  | 1.26 (1.19, 1.34) | <0.001 |
| MAF-5 | 1092 (6.82) | 1.52 (1.43, 1.62) | <0.001 |  | 1.33 (1.24, 1.42) | <0.001 |  | 1.34 (1.23, 1.46) | <0.001 |
| NFS | 1092 (6.82) | 2.05 (1.81, 2.33) | <0.001 |  | 1.83 (1.61, 2.08) | <0.001 |  | 1.60 (1.43, 1.79) | <0.001 |
| FIB-4 | 1092 (6.82) | 1.24 (1.17, 1.32) | <0.001 |  | 1.25 (1.19, 1.31) | <0.001 |  | 1.21 (1.16, 1.26) | <0.001 |
| SAFE | 1092 (6.82) | 2.19 (2.05, 2.34) | <0.001 |  | 2.02 (1.88, 2.16) | <0.001 |  | 1.82 (1.67, 1.98) | <0.001 |
| APRI | 1092 (6.82) | 1.13 (1.10, 1.17) | <0.001 |  | 1.13 (1.09, 1.17) | <0.001 |  | 1.12 (1.09, 1.16) | <0.001 |
| Categorical variables |  |  |  |  |  |  |  |  |  |
| LiverRisk |  |  |  |  |  |  |  |  |  |
| Low | 541 (4.77) | 1.00 (Reference) |  |  | 1.00 (Reference) |  |  | 1.00 (Reference) |  |
| Intermediate | 474 (12.51) | 3.23 (2.76, 3.78) | <0.001 |  | 2.90 (2.46, 3.43) | <0.001 |  | 2.13 (1.80, 2.53) | <0.001 |
| High | 77 (28.53) | 8.17 (5.93, 11.26) | <0.001 |  | 6.52 (4.87, 8.74) | <0.001 |  | 4.96 (3.56, 6.91) | <0.001 |
| *P* for trend |  | <0.001 |  |  | <0.001 |  |  | <0.001 |  |
| MAF-5 |  |  |  |  |  |  |  |  |  |
| Low | 364 (4.54) | 1.00 (Reference) |  |  | 1.00 (Reference) |  |  | 1.00 (Reference) |  |
| Intermediate | 181 (6.65) | 1.50 (1.22, 1.84) | <0.001 |  | 1.09 (0.88, 1.35) | 0.413 |  | 1.10 (0.88, 1.37) | 0.399 |
| High | 547 (11.06) | 2.75 (2.36, 3.20) | <0.001 |  | 1.66 (1.42, 1.93) | <0.001 |  | 1.64 (1.36, 1.99) | <0.001 |
| *P* for trend |  | <0.001 |  |  | <0.001 |  |  | <0.001 |  |
| NFS |  |  |  |  |  |  |  |  |  |
| Low | 207 (3.45) | 1.00 (Reference) |  |  | 1.00 (Reference) |  |  | 1.00 (Reference) |  |
| Intermediate | 546 (7.27) | 2.66 (2.16, 3.28) | <0.001 |  | 2.42 (1.95, 3.00) | <0.001 |  | 1.88 (1.52, 2.33) | <0.001 |
| High | 339 (21.14) | 9.73 (7.57, 12.52) | <0.001 |  | 7.08 (5.47, 9.14) | <0.001 |  | 4.43 (3.40, 5.78) | <0.001 |
| *P* for trend |  | <0.001 |  |  | <0.001 |  |  | <0.001 |  |
| FIB-4 |  |  |  |  |  |  |  |  |  |
| Low | 674 (5.66) | 1.00 (Reference) |  |  | 1.00 (Reference) |  |  | 1.00 (Reference) |  |
| Intermediate | 280 (8.26) | 1.70 (1.43, 2.02) | <0.001 |  | 1.67 (1.40, 1.99) | <0.001 |  | 1.36 (1.13, 1.64) | 0.001 |
| High | 138 (31.53) | 8.29 (6.41, 10.73) | <0.001 |  | 6.45 (4.98, 8.35) | <0.001 |  | 3.93 (3.02, 5.11) | <0.001 |
| *P* for trend |  | <0.001 |  |  | <0.001 |  |  | <0.001 |  |
| SAFE |  |  |  |  |  |  |  |  |  |
| Low | 185 (2.89) | 1.00 (Reference) |  |  | 1.00 (Reference) |  |  | 1.00 (Reference) |  |
| Intermediate | 394 (7.92) | 3.02 (2.41, 3.78) | <0.001 |  | 2.73 (2.15, 3.46) | <0.001 |  | 2.20 (1.72, 2.81) | <0.001 |
| High | 513 (20.17) | 9.11 (7.34, 11.31) | <0.001 |  | 6.92 (5.53, 8.64) | <0.001 |  | 4.71 (3.62, 6.12) | <0.001 |
| *P* for trend |  | <0.001 |  |  | <0.001 |  |  | <0.001 |  |
| APRI |  |  |  |  |  |  |  |  |  |
| Low | 1036 (6.55) | 1.00 (Reference) |  |  | 1.00 (Reference) |  |  | 1.00 (Reference) |  |
| High | 56 (23.36) | 4.67 (3.22, 6.77) | <0.001 |  | 3.68 (2.49, 5.41) | <0.001 |  | 3.37 (2.32, 4.90) | <0.001 |

Model 1: Unadjusted.

Model 2: Adjusted for age, sex, race/ethnicity, education level, family PIR, marital status, smoking, drinking, and physical activity.

Model 3: Adjusted for Model 2 variables plus BMI, eGFR, hypertension, diabetes, hyperlipidemia, and NAFLD.

Note: For each fibrosis score, variables used in the calculation of that score were not additionally included in the corresponding multivariable model, as described in the Methods.

APRI, aspartate aminotransferase to platelet ratio index score; BMI, body mass index; CI, confidence interval; CKM, cardiovascular-kidney-metabolic; eGFR, estimated glomerular filtration rate; FIB-4, Fibrosis-4 index score; LiverRisk, LiverRisk score; MAF-5, metabolic dysfunction-associated fibrosis-5 score; NAFLD, nonalcoholic fatty liver disease; NFS, nonalcoholic fatty liver disease fibrosis score; PIR, poverty income ratio; SAFE, steatosis-associated fibrosis estimator score; SD, standard deviation; HR, hazard ratio.

**Table S9. Associations between noninvasive liver fibrosis scores and CVD mortality among participants with CKM syndrome (after multiple imputation)**

| **Noninvasive liver fibrosis score** | **Event, n (%)** | **Model 1** | |  | **Model 2** | |  | **Model 3** | |
| --- | --- | --- | --- | --- | --- | --- | --- | --- | --- |
|  |  | ***sHR* (95% *CI*)** | ***P*** |  | ***sHR* (95% *CI*)** | ***P*** |  | ***sHR* (95% *CI*)** | ***P*** |
| Continuous variables (per 1 SD increase) |  |  |  |  |  |  |  |  |  |
| LiverRisk | 314 (1.84) | 1.31 (1.23, 1.41) | <0.001 |  | 1.28 (1.20, 1.37) | <0.001 |  | 1.20 (1.10, 1.32) | <0.001 |
| MAF-5 | 314 (1.84) | 1.58 (1.45, 1.72) | <0.001 |  | 1.34 (1.21, 1.48) | <0.001 |  | 1.23 (1.06, 1.43) | 0.008 |
| NFS | 314 (1.84) | 2.21 (1.88, 2.59) | <0.001 |  | 1.92 (1.66, 2.23) | <0.001 |  | 1.61 (1.42, 1.83) | <0.001 |
| FIB-4 | 314 (1.84) | 1.22 (1.15, 1.28) | <0.001 |  | 1.19 (1.12, 1.26) | <0.001 |  | 1.15 (1.08, 1.22) | <0.001 |
| SAFE | 314 (1.84) | 2.17 (2.02, 2.34) | <0.001 |  | 1.92 (1.76, 2.10) | <0.001 |  | 1.63 (1.45, 1.82) | <0.001 |
| APRI | 314 (1.84) | 1.09 (1.05, 1.14) | <0.001 |  | 1.05 (0.98, 1.13) | 0.181 |  | 1.06 (0.98, 1.14) | 0.142 |
| Categorical variables |  |  |  |  |  |  |  |  |  |
| LiverRisk |  |  |  |  |  |  |  |  |  |
| Low | 148 (1.23) | 1.00 (Reference) |  |  | 1.00 (Reference) |  |  | 1.00 (Reference) |  |
| Intermediate | 142 (3.59) | 3.45 (2.72, 4.38) | <0.001 |  | 3.05 (2.38, 3.90) | <0.001 |  | 1.84 (1.33, 2.56) | <0.001 |
| High | 24 (7.78) | 7.30 (4.69, 11.36) | <0.001 |  | 5.35 (3.27, 8.75) | <0.001 |  | 3.32 (1.77, 6.23) | <0.001 |
| *P* for trend |  | <0.001 |  |  | <0.001 |  |  | <0.001 |  |
| MAF-5 |  |  |  |  |  |  |  |  |  |
| Low | 88 (0.92) | 1.00 (Reference) |  |  | 1.00 (Reference) |  |  | 1.00 (Reference) |  |
| Intermediate | 47 (1.95) | 2.16 (1.43, 3.26) | <0.001 |  | 1.46 (0.94, 2.26) | 0.094 |  | 1.33 (0.81, 2.19) | 0.267 |
| High | 179 (3.46) | 4.09 (2.99, 5.60) | <0.001 |  | 2.22 (1.60, 3.06) | <0.001 |  | 1.86 (1.17, 2.96) | 0.009 |
| *P* for trend |  | <0.001 |  |  | <0.001 |  |  | 0.008 |  |
| NFS |  |  |  |  |  |  |  |  |  |
| Low | 45 (0.71) | 1.00 (Reference) |  |  | 1.00 (Reference) |  |  | 1.00 (Reference) |  |
| Intermediate | 164 (2.01) | 3.48 (2.20, 5.50) | <0.001 |  | 3.05 (1.93, 4.83) | <0.001 |  | 2.01 (1.22, 3.30) | 0.006 |
| High | 105 (6.57) | 12.79 (8.31, 19.68) | <0.001 |  | 8.44 (5.51, 12.94) | <0.001 |  | 3.93 (2.48, 6.22) | <0.001 |
| *P* for trend |  | <0.001 |  |  | <0.001 |  |  | <0.001 |  |
| FIB-4 |  |  |  |  |  |  |  |  |  |
| Low | 195 (1.56) | 1.00 (Reference) |  |  | 1.00 (Reference) |  |  | 1.00 (Reference) |  |
| Intermediate | 88 (2.35) | 1.71 (1.26, 2.31) | <0.001 |  | 1.58 (1.15, 2.16) | 0.005 |  | 1.22 (0.88, 1.69) | 0.237 |
| High | 31 (6.73) | 5.15 (3.25, 8.18) | <0.001 |  | 3.53 (2.16, 5.76) | <0.001 |  | 2.07 (1.26, 3.40) | 0.004 |
| *P* for trend |  | <0.001 |  |  | <0.001 |  |  | 0.007 |  |
| SAFE |  |  |  |  |  |  |  |  |  |
| Low | 38 (0.54) | 1.00 (Reference) |  |  | 1.00 (Reference) |  |  | 1.00 (Reference) |  |
| Intermediate | 121 (2.37) | 4.79 (2.93, 7.82) | <0.001 |  | 4.10 (2.48, 6.77) | <0.001 |  | 2.71 (1.57, 4.67) | <0.001 |
| High | 155 (5.87) | 12.75 (8.28, 19.63) | <0.001 |  | 8.63 (5.55, 13.44) | <0.001 |  | 4.36 (2.62, 7.27) | <0.001 |
| *P* for trend |  | <0.001 |  |  | <0.001 |  |  | <0.001 |  |
| APRI |  |  |  |  |  |  |  |  |  |
| Low | 304 (1.80) | 1.00 (Reference) |  |  | 1.00 (Reference) |  |  | 1.00 (Reference) |  |
| High | 10 (4.51) | 2.89 (1.45, 5.75) | 0.002 |  | 2.06 (1.04, 4.06) | 0.038 |  | 2.01 (0.99, 4.09) | 0.052 |

Model 1: Unadjusted.

Model 2: Adjusted for age, sex, race/ethnicity, education level, family PIR, marital status, smoking, drinking, and physical activity.

Model 3: Adjusted for Model 2 variables plus BMI, eGFR, hypertension, diabetes, hyperlipidemia, and NAFLD.

Note: For each fibrosis score, variables used in the calculation of that score were not additionally included in the corresponding multivariable model, as described in the Methods.

APRI, aspartate aminotransferase to platelet ratio index score; BMI, body mass index; CI, confidence interval; CKM, cardiovascular-kidney-metabolic; eGFR, estimated glomerular filtration rate; FIB-4, Fibrosis-4 index score; LiverRisk, LiverRisk score; MAF-5, metabolic dysfunction-associated fibrosis-5 score; NAFLD, nonalcoholic fatty liver disease; NFS, nonalcoholic fatty liver disease fibrosis score; PIR, poverty income ratio; SAFE, steatosis-associated fibrosis estimator score; SD, standard deviation; sHR, subdistribution hazard ratio.

**Table S10. Associations between noninvasive liver fibrosis scores and all-cause mortality among participants with CKM syndrome (excluding accidental mortality)**

| **Noninvasive liver fibrosis score** | **Event, n (%)** | **Model 1** | |  | **Model 2** | |  | **Model 3** | |
| --- | --- | --- | --- | --- | --- | --- | --- | --- | --- |
|  |  | ***HR* (95% *CI*)** | ***P*** |  | ***HR* (95% *CI*)** | ***P*** |  | ***HR* (95% *CI*)** | ***P*** |
| Continuous variables (per 1 SD increase) |  |  |  |  |  |  |  |  |  |
| LiverRisk | 582 (5.14) | 1.30 (1.23, 1.38) | <0.001 |  | 1.26 (1.19, 1.33) | <0.001 |  | 1.25 (1.18, 1.33) | <0.001 |
| MAF-5 | 582 (5.14) | 1.51 (1.38, 1.65) | <0.001 |  | 1.29 (1.15, 1.45) | <0.001 |  | 1.30 (1.12, 1.51) | <0.001 |
| NFS | 582 (5.14) | 2.21 (1.94, 2.51) | <0.001 |  | 2.02 (1.78, 2.30) | <0.001 |  | 1.77 (1.52, 2.05) | <0.001 |
| FIB-4 | 582 (5.14) | 1.31 (1.26, 1.37) | <0.001 |  | 1.27 (1.21, 1.34) | <0.001 |  | 1.23 (1.17, 1.30) | <0.001 |
| SAFE | 582 (5.14) | 2.10 (1.92, 2.29) | <0.001 |  | 1.94 (1.78, 2.12) | <0.001 |  | 1.79 (1.61, 1.99) | <0.001 |
| APRI | 582 (5.14) | 1.14 (1.10, 1.18) | <0.001 |  | 1.14 (1.10, 1.18) | <0.001 |  | 1.13 (1.09, 1.17) | <0.001 |
| Categorical variables |  |  |  |  |  |  |  |  |  |
| LiverRisk |  |  |  |  |  |  |  |  |  |
| Low | 314 (3.67) | 1.00 (Reference) |  |  | 1.00 (Reference) |  |  | 1.00 (Reference) |  |
| Intermediate | 232 (9.46) | 3.29 (2.65, 4.09) | <0.001 |  | 2.91 (2.30, 3.68) | <0.001 |  | 2.25 (1.73, 2.93) | <0.001 |
| High | 36 (24.36) | 9.37 (5.48, 16.03) | <0.001 |  | 7.68 (4.64, 12.71) | <0.001 |  | 6.68 (4.06, 10.97) | <0.001 |
| *P* for trend |  | <0.001 |  |  | <0.001 |  |  | <0.001 |  |
| MAF-5 |  |  |  |  |  |  |  |  |  |
| Low | 205 (3.48) | 1.00 (Reference) |  |  | 1.00 (Reference) |  |  | 1.00 (Reference) |  |
| Intermediate | 101 (5.50) | 1.67 (1.26, 2.20) | <0.001 |  | 1.21 (0.92, 1.59) | 0.172 |  | 1.23 (0.91, 1.66) | 0.180 |
| High | 276 (8.13) | 2.71 (2.18, 3.37) | <0.001 |  | 1.64 (1.29, 2.08) | <0.001 |  | 1.65 (1.21, 2.24) | 0.002 |
| *P* for trend |  | <0.001 |  |  | <0.001 |  |  | 0.002 |  |
| NFS |  |  |  |  |  |  |  |  |  |
| Low | 117 (2.66) | 1.00 (Reference) |  |  | 1.00 (Reference) |  |  | 1.00 (Reference) |  |
| Intermediate | 305 (5.63) | 2.77 (2.08, 3.68) | <0.001 |  | 2.54 (1.88, 3.42) | <0.001 |  | 2.04 (1.50, 2.78) | <0.001 |
| High | 160 (16.38) | 10.26 (7.32, 14.38) | <0.001 |  | 7.82 (5.62, 10.89) | <0.001 |  | 5.20 (3.54, 7.64) | <0.001 |
| *P* for trend |  | <0.001 |  |  | <0.001 |  |  | <0.001 |  |
| FIB-4 |  |  |  |  |  |  |  |  |  |
| Low | 356 (4.14) | 1.00 (Reference) |  |  | 1.00 (Reference) |  |  | 1.00 (Reference) |  |
| Intermediate | 148 (6.36) | 1.84 (1.45, 2.33) | <0.001 |  | 1.76 (1.38, 2.24) | <0.001 |  | 1.44 (1.14, 1.83) | 0.002 |
| High | 78 (29.16) | 11.19 (7.87, 15.92) | <0.001 |  | 8.95 (6.33, 12.65) | <0.001 |  | 5.77 (4.07, 8.17) | <0.001 |
| *P* for trend |  | <0.001 |  |  | <0.001 |  |  | <0.001 |  |
| SAFE |  |  |  |  |  |  |  |  |  |
| Low | 111 (2.40) | 1.00 (Reference) |  |  | 1.00 (Reference) |  |  | 1.00 (Reference) |  |
| Intermediate | 215 (5.68) | 2.68 (2.01, 3.58) | <0.001 |  | 2.39 (1.77, 3.23) | <0.001 |  | 1.96 (1.42, 2.70) | <0.001 |
| High | 256 (16.76) | 9.72 (7.27, 13.00) | <0.001 |  | 7.38 (5.45, 10.00) | <0.001 |  | 5.22 (3.63, 7.50) | <0.001 |
| *P* for trend |  | <0.001 |  |  | <0.001 |  |  | <0.001 |  |
| APRI |  |  |  |  |  |  |  |  |  |
| Low | 547 (4.87) | 1.00 (Reference) |  |  | 1.00 (Reference) |  |  | 1.00 (Reference) |  |
| High | 35 (22.52) | 6.00 (3.73, 9.65) | <0.001 |  | 4.29 (2.64, 6.98) | <0.001 |  | 3.86 (2.42, 6.17) | <0.001 |

Model 1: Unadjusted.

Model 2: Adjusted for age, sex, race/ethnicity, education level, family PIR, marital status, smoking, drinking, and physical activity.

Model 3: Adjusted for Model 2 variables plus BMI, eGFR, hypertension, diabetes, hyperlipidemia, and NAFLD.

Note: For each fibrosis score, variables used in the calculation of that score were not additionally included in the corresponding multivariable model, as described in the Methods.

APRI, aspartate aminotransferase to platelet ratio index score; BMI, body mass index; CI, confidence interval; CKM, cardiovascular-kidney-metabolic; eGFR, estimated glomerular filtration rate; FIB-4, Fibrosis-4 index score; LiverRisk, LiverRisk score; MAF-5, metabolic dysfunction-associated fibrosis-5 score; NAFLD, nonalcoholic fatty liver disease; NFS, nonalcoholic fatty liver disease fibrosis score; PIR, poverty income ratio; SAFE, steatosis-associated fibrosis estimator score; SD, standard deviation; HR, hazard ratio.

**Table S11. Associations between noninvasive liver fibrosis scores and CVD mortality among participants with CKM syndrome (excluding accidental mortality)**

| **Noninvasive liver fibrosis score** | **Event, n (%)** | **Model 1** | |  | **Model 2** | |  | **Model 3** | |
| --- | --- | --- | --- | --- | --- | --- | --- | --- | --- |
|  |  | ***sHR* (95% *CI*)** | ***P*** |  | ***sHR* (95% *CI*)** | ***P*** |  | ***sHR* (95% *CI*)** | ***P*** |
| Continuous variables (per 1 SD increase) |  |  |  |  |  |  |  |  |  |
| LiverRisk | 166 (1.43) | 1.25 (1.16, 1.35) | <0.001 |  | 1.22 (1.14, 1.30) | <0.001 |  | 1.20 (1.10, 1.31) | <0.001 |
| MAF-5 | 166 (1.43) | 1.52 (1.32, 1.73) | <0.001 |  | 1.20 (1.01, 1.43) | 0.037 |  | 1.11 (0.87, 1.43) | 0.404 |
| NFS | 166 (1.43) | 2.31 (1.89, 2.83) | <0.001 |  | 1.99 (1.64, 2.42) | <0.001 |  | 1.63 (1.32, 2.02) | <0.001 |
| FIB-4 | 166 (1.43) | 1.23 (1.14, 1.33) | <0.001 |  | 1.19 (1.10, 1.28) | <0.001 |  | 1.13 (1.04, 1.24) | 0.004 |
| SAFE | 166 (1.43) | 1.99 (1.79, 2.22) | <0.001 |  | 1.76 (1.56, 1.98) | <0.001 |  | 1.51 (1.28, 1.77) | <0.001 |
| APRI | 166 (1.43) | 1.10 (1.04, 1.16) | 0.001 |  | 1.06 (0.97, 1.16) | 0.19 |  | 1.05 (0.96, 1.16) | 0.277 |
| Categorical variables |  |  |  |  |  |  |  |  |  |
| LiverRisk |  |  |  |  |  |  |  |  |  |
| Low | 94 (1.10) | 1.00 (Reference) |  |  | 1.00 (Reference) |  |  | 1.00 (Reference) |  |
| Intermediate | 60 (2.38) | 2.71 (1.86, 3.94) | <0.001 |  | 2.32 (1.58, 3.39) | <0.001 |  | 1.53 (0.88, 2.64) | 0.130 |
| High | 12 (6.06) | 6.75 (3.27, 13.93) | <0.001 |  | 4.94 (2.34, 10.43) | <0.001 |  | 3.88 (1.56, 9.64) | 0.004 |
| *P* for trend |  | <0.001 |  |  | <0.001 |  |  | 0.020 |  |
| MAF-5 |  |  |  |  |  |  |  |  |  |
| Low | 58 (0.90) | 1.00 (Reference) |  |  | 1.00 (Reference) |  |  | 1.00 (Reference) |  |
| Intermediate | 26 (1.47) | 1.74 (0.97, 3.11) | 0.061 |  | 1.08 (0.59, 1.98) | 0.791 |  | 1.01 (0.49, 2.09) | 0.971 |
| High | 82 (2.44) | 3.12 (2.01, 4.85) | <0.001 |  | 1.60 (1.03, 2.50) | 0.038 |  | 1.38 (0.72, 2.68) | 0.333 |
| *P* for trend |  | <0.001 |  |  | 0.038 |  |  | 0.314 |  |
| NFS |  |  |  |  |  |  |  |  |  |
| Low | 28 (0.64) | 1.00 (Reference) |  |  | 1.00 (Reference) |  |  | 1.00 (Reference) |  |
| Intermediate | 92 (1.69) | 3.49 (1.88, 6.48) | <0.001 |  | 3.02 (1.64, 5.59) | <0.001 |  | 2.16 (1.11, 4.23) | 0.024 |
| High | 46 (4.45) | 10.73 (5.14, 22.39) | <0.001 |  | 6.78 (3.29, 13.97) | <0.001 |  | 3.56 (1.66, 7.64) | 0.001 |
| *P* for trend |  | <0.001 |  |  | <0.001 |  |  | <0.001 |  |
| FIB-4 |  |  |  |  |  |  |  |  |  |
| Low | 106 (1.23) | 1.00 (Reference) |  |  | 1.00 (Reference) |  |  | 1.00 (Reference) |  |
| Intermediate | 43 (1.79) | 1.76 (1.12, 2.77) | 0.014 |  | 1.56 (0.96, 2.53) | 0.07 |  | 1.18 (0.74, 1.90) | 0.488 |
| High | 17 (5.26) | 5.43 (2.98, 9.89) | <0.001 |  | 3.64 (1.93, 6.87) | <0.001 |  | 2.27 (1.18, 4.38) | 0.014 |
| *P* for trend |  | <0.001 |  |  | <0.001 |  |  | 0.063 |  |
| SAFE |  |  |  |  |  |  |  |  |  |
| Low | 28 (0.60) | 1.00 (Reference) |  |  | 1.00 (Reference) |  |  | 1.00 (Reference) |  |
| Intermediate | 61 (1.66) | 3.11 (1.69, 5.74) | <0.001 |  | 2.57 (1.39, 4.76) | 0.003 |  | 1.80 (0.91, 3.56) | 0.093 |
| High | 77 (4.74) | 10.10 (5.63, 18.11) | <0.001 |  | 6.52 (3.58, 11.88) | <0.001 |  | 3.65 (1.78, 7.48) | <0.001 |
| *P* for trend |  | <0.001 |  |  | <0.001 |  |  | <0.001 |  |
| APRI |  |  |  |  |  |  |  |  |  |
| Low | 159 (1.39) | 1.00 (Reference) |  |  | 1.00 (Reference) |  |  | 1.00 (Reference) |  |
| High | 7 (4.11) | 3.39 (1.46, 7.88) | 0.004 |  | 2.13 (0.88, 5.13) | 0.092 |  | 1.94 (0.75, 4.99) | 0.172 |

Model 1: Unadjusted.

Model 2: Adjusted for age, sex, race/ethnicity, education level, family PIR, marital status, smoking, drinking, and physical activity.

Model 3: Adjusted for Model 2 variables plus BMI, eGFR, hypertension, diabetes, hyperlipidemia, and NAFLD.

Note: For each fibrosis score, variables used in the calculation of that score were not additionally included in the corresponding multivariable model, as described in the Methods.

APRI, aspartate aminotransferase to platelet ratio index score; BMI, body mass index; CI, confidence interval; CKM, cardiovascular-kidney-metabolic; eGFR, estimated glomerular filtration rate; FIB-4, Fibrosis-4 index score; LiverRisk, LiverRisk score; MAF-5, metabolic dysfunction-associated fibrosis-5 score; NAFLD, nonalcoholic fatty liver disease; NFS, nonalcoholic fatty liver disease fibrosis score; PIR, poverty income ratio; SAFE, steatosis-associated fibrosis estimator score; SD, standard deviation; sHR, subdistribution hazard ratio.

**Table S12. Associations between noninvasive liver fibrosis scores and all-cause mortality among participants with CKM syndrome (excluding events within two years)**

| **Noninvasive liver fibrosis score** | **Event, n (%)** | **Model 1** | |  | **Model 2** | |  | **Model 3** | |
| --- | --- | --- | --- | --- | --- | --- | --- | --- | --- |
|  |  | ***HR* (95% *CI*)** | ***P*** |  | ***HR* (95% *CI*)** | ***P*** |  | ***HR* (95% *CI*)** | ***P*** |
| Continuous variables (per 1 SD increase) |  |  |  |  |  |  |  |  |  |
| LiverRisk | 504 (4.92) | 1.31 (1.23, 1.39) | <0.001 |  | 1.28 (1.20, 1.35) | <0.001 |  | 1.27 (1.19, 1.35) | <0.001 |
| MAF-5 | 504 (4.92) | 1.54 (1.40, 1.68) | <0.001 |  | 1.32 (1.18, 1.48) | <0.001 |  | 1.34 (1.17, 1.53) | <0.001 |
| NFS | 504 (4.92) | 2.28 (2.02, 2.58) | <0.001 |  | 2.09 (1.84, 2.37) | <0.001 |  | 1.82 (1.59, 2.10) | <0.001 |
| FIB-4 | 504 (4.92) | 1.32 (1.27, 1.37) | <0.001 |  | 1.28 (1.22, 1.34) | <0.001 |  | 1.23 (1.18, 1.29) | <0.001 |
| SAFE | 504 (4.92) | 2.13 (1.95, 2.34) | <0.001 |  | 1.99 (1.82, 2.19) | <0.001 |  | 1.82 (1.63, 2.03) | <0.001 |
| APRI | 504 (4.92) | 1.14 (1.10, 1.19) | <0.001 |  | 1.15 (1.12, 1.18) | <0.001 |  | 1.14 (1.10, 1.17) | <0.001 |
| Categorical variables |  |  |  |  |  |  |  |  |  |
| LiverRisk |  |  |  |  |  |  |  |  |  |
| Low | 272 (3.44) | 1.00 (Reference) |  |  | 1.00 (Reference) |  |  | 1.00 (Reference) |  |
| Intermediate | 201 (9.59) | 3.58 (2.88, 4.45) | <0.001 |  | 3.23 (2.54, 4.11) | <0.001 |  | 2.53 (1.93, 3.31) | <0.001 |
| High | 31 (22.28) | 9.71 (5.43, 17.36) | <0.001 |  | 8.18 (4.72, 14.16) | <0.001 |  | 7.15 (4.32, 11.85) | <0.001 |
| *P* for trend |  | <0.001 |  |  | <0.001 |  |  | <0.001 |  |
| MAF-5 |  |  |  |  |  |  |  |  |  |
| Low | 169 (3.17) | 1.00 (Reference) |  |  | 1.00 (Reference) |  |  | 1.00 (Reference) |  |
| Intermediate | 93 (5.47) | 1.89 (1.43, 2.51) | <0.001 |  | 1.38 (1.03, 1.84) | 0.029 |  | 1.43 (1.06, 1.94) | 0.020 |
| High | 242 (7.98) | 2.99 (2.39, 3.73) | <0.001 |  | 1.82 (1.44, 2.30) | <0.001 |  | 1.90 (1.44, 2.51) | <0.001 |
| *P* for trend |  | <0.001 |  |  | <0.001 |  |  | <0.001 |  |
| NFS |  |  |  |  |  |  |  |  |  |
| Low | 99 (2.37) | 1.00 (Reference) |  |  | 1.00 (Reference) |  |  | 1.00 (Reference) |  |
| Intermediate | 269 (5.70) | 3.11 (2.35, 4.12) | <0.001 |  | 2.87 (2.12, 3.87) | <0.001 |  | 2.29 (1.69, 3.12) | <0.001 |
| High | 136 (16.16) | 11.41 (8.06, 16.13) | <0.001 |  | 8.66 (6.21, 12.09) | <0.001 |  | 5.75 (3.95, 8.37) | <0.001 |
| *P* for trend |  | <0.001 |  |  | <0.001 |  |  | <0.001 |  |
| FIB-4 |  |  |  |  |  |  |  |  |  |
| Low | 312 (4.05) | 1.00 (Reference) |  |  | 1.00 (Reference) |  |  | 1.00 (Reference) |  |
| Intermediate | 127 (6.02) | 1.85 (1.44, 2.37) | <0.001 |  | 1.80 (1.39, 2.32) | <0.001 |  | 1.45 (1.13, 1.87) | 0.004 |
| High | 65 (26.93) | 10.80 (7.70, 15.15) | <0.001 |  | 9.04 (6.43, 12.71) | <0.001 |  | 5.63 (4.02, 7.87) | <0.001 |
| *P* for trend |  | <0.001 |  |  | <0.001 |  |  | <0.001 |  |
| SAFE |  |  |  |  |  |  |  |  |  |
| Low | 94 (2.21) | 1.00 (Reference) |  |  | 1.00 (Reference) |  |  | 1.00 (Reference) |  |
| Intermediate | 185 (5.69) | 2.91 (2.14, 3.95) | <0.001 |  | 2.61 (1.90, 3.58) | <0.001 |  | 2.14 (1.53, 2.99) | <0.001 |
| High | 225 (16.15) | 10.56 (7.83, 14.25) | <0.001 |  | 8.18 (6.00, 11.14) | <0.001 |  | 5.72 (3.94, 8.30) | <0.001 |
| *P* for trend |  | <0.001 |  |  | <0.001 |  |  | <0.001 |  |
| APRI |  |  |  |  |  |  |  |  |  |
| Low | 474 (4.67) | 1.00 (Reference) |  |  | 1.00 (Reference) |  |  | 1.00 (Reference) |  |
| High | 30 (22.23) | 6.04 (3.58, 10.19) | <0.001 |  | 4.58 (2.74, 7.64) | <0.001 |  | 4.01 (2.49, 6.45) | <0.001 |

Model 1: Unadjusted.

Model 2: Adjusted for age, sex, race/ethnicity, education level, family PIR, marital status, smoking, drinking, and physical activity.

Model 3: Adjusted for Model 2 variables plus BMI, eGFR, hypertension, diabetes, hyperlipidemia, and NAFLD.

Note: For each fibrosis score, variables used in the calculation of that score were not additionally included in the corresponding multivariable model, as described in the Methods.

APRI, aspartate aminotransferase to platelet ratio index score; BMI, body mass index; CI, confidence interval; CKM, cardiovascular-kidney-metabolic; eGFR, estimated glomerular filtration rate; FIB-4, Fibrosis-4 index score; LiverRisk, LiverRisk score; MAF-5, metabolic dysfunction-associated fibrosis-5 score; NAFLD, nonalcoholic fatty liver disease; NFS, nonalcoholic fatty liver disease fibrosis score; PIR, poverty income ratio; SAFE, steatosis-associated fibrosis estimator score; SD, standard deviation; HR, hazard ratio.

**Table S13. Associations between noninvasive liver fibrosis scores and CVD mortality among participants with CKM syndrome (excluding events within two years)**

| **Noninvasive liver fibrosis score** | **Event, n (%)** | **Model 1** | |  | **Model 2** | |  | **Model 3** | |
| --- | --- | --- | --- | --- | --- | --- | --- | --- | --- |
|  |  | ***sHR* (95% *CI*)** | ***P*** |  | ***sHR* (95% *CI*)** | ***P*** |  | ***sHR* (95% *CI*)** | ***P*** |
| Continuous variables (per 1 SD increase) |  |  |  |  |  |  |  |  |  |
| LiverRisk | 146 (1.37) | 1.26 (1.16, 1.36) | <0.001 |  | 1.23 (1.14, 1.33) | <0.001 |  | 1.21 (1.09, 1.34) | <0.001 |
| MAF-5 | 146 (1.37) | 1.58 (1.38, 1.81) | <0.001 |  | 1.28 (1.08, 1.52) | 0.005 |  | 1.23 (0.97, 1.55) | 0.089 |
| NFS | 146 (1.37) | 2.47 (2.01, 3.04) | <0.001 |  | 2.11 (1.71, 2.60) | <0.001 |  | 1.72 (1.36, 2.17) | <0.001 |
| FIB-4 | 146 (1.37) | 1.23 (1.14, 1.33) | <0.001 |  | 1.20 (1.12, 1.30) | <0.001 |  | 1.15 (1.05, 1.26) | 0.003 |
| SAFE | 146 (1.37) | 2.10 (1.87, 2.34) | <0.001 |  | 1.86 (1.63, 2.11) | <0.001 |  | 1.59 (1.33, 1.91) | <0.001 |
| APRI | 146 (1.37) | 1.11 (1.05, 1.17) | <0.001 |  | 1.08 (0.99, 1.17) | 0.083 |  | 1.07 (0.98, 1.18) | 0.147 |
| Categorical variables |  |  |  |  |  |  |  |  |  |
| LiverRisk |  |  |  |  |  |  |  |  |  |
| Low | 80 (0.99) | 1.00 (Reference) |  |  | 1.00 (Reference) |  |  | 1.00 (Reference) |  |
| Intermediate | 55 (2.55) | 3.23 (2.19, 4.77) | <0.001 |  | 2.79 (1.86, 4.17) | <0.001 |  | 1.84 (1.01, 3.36) | 0.047 |
| High | 11 (6.13) | 8.07 (3.81, 17.11) | <0.001 |  | 5.89 (2.63, 13.18) | <0.001 |  | 4.62 (1.69, 12.66) | 0.003 |
| *P* for trend |  | <0.001 |  |  | <0.001 |  |  | 0.006 |  |
| MAF-5 |  |  |  |  |  |  |  |  |  |
| Low | 49 (0.77) | 1.00 (Reference) |  |  | 1.00 (Reference) |  |  | 1.00 (Reference) |  |
| Intermediate | 25 (1.56) | 2.24 (1.28, 3.91) | 0.005 |  | 1.37 (0.77, 2.47) | 0.286 |  | 1.32 (0.66, 2.61) | 0.430 |
| High | 72 (2.42) | 3.69 (2.44, 5.59) | <0.001 |  | 1.90 (1.22, 2.95) | 0.004 |  | 1.78 (0.99, 3.22) | 0.054 |
| *P* for trend |  | <0.001 |  |  | 0.005 |  |  | 0.052 |  |
| NFS |  |  |  |  |  |  |  |  |  |
| Low | 24 (0.58) | 1.00 (Reference) |  |  | 1.00 (Reference) |  |  | 1.00 (Reference) |  |
| Intermediate | 79 (1.57) | 3.50 (1.82, 6.70) | <0.001 |  | 3.05 (1.58, 5.89) | <0.001 |  | 2.18 (1.06, 4.49) | 0.034 |
| High | 43 (5.07) | 13.40 (6.41, 28.01) | <0.001 |  | 8.16 (3.81, 17.48) | <0.001 |  | 4.19 (1.86, 9.48) | <0.001 |
| *P* for trend |  | <0.001 |  |  | <0.001 |  |  | <0.001 |  |
| FIB-4 |  |  |  |  |  |  |  |  |  |
| Low | 94 (1.18) | 1.00 (Reference) |  |  | 1.00 (Reference) |  |  | 1.00 (Reference) |  |
| Intermediate | 36 (1.61) | 1.70 (1.05, 2.75) | 0.03 |  | 1.52 (0.93, 2.48) | 0.093 |  | 1.09 (0.66, 1.81) | 0.739 |
| High | 16 (6.08) | 6.79 (3.67, 12.53) | <0.001 |  | 4.64 (2.40, 8.98) | <0.001 |  | 2.77 (1.36, 5.66) | 0.005 |
| *P* for trend |  | <0.001 |  |  | <0.001 |  |  | 0.064 |  |
| SAFE |  |  |  |  |  |  |  |  |  |
| Low | 22 (0.48) | 1.00 (Reference) |  |  | 1.00 (Reference) |  |  | 1.00 (Reference) |  |
| Intermediate | 53 (1.63) | 3.81 (1.89, 7.71) | <0.001 |  | 3.14 (1.55, 6.34) | 0.001 |  | 2.20 (1.03, 4.68) | 0.041 |
| High | 71 (5.03) | 13.95 (7.47, 26.07) | <0.001 |  | 9.00 (4.82, 16.81) | <0.001 |  | 5.06 (2.45, 10.42) | <0.001 |
| *P* for trend |  | <0.001 |  |  | <0.001 |  |  | <0.001 |  |
| APRI |  |  |  |  |  |  |  |  |  |
| Low | 139 (1.32) | 1.00 (Reference) |  |  | 1.00 (Reference) |  |  | 1.00 (Reference) |  |
| High | 7 (4.91) | 4.19 (1.83, 9.64) | <0.001 |  | 2.60 (1.04, 6.50) | 0.041 |  | 2.32 (0.82, 6.56) | 0.114 |

Model 1: Unadjusted.

Model 2: Adjusted for age, sex, race/ethnicity, education level, family PIR, marital status, smoking, drinking, and physical activity.

Model 3: Adjusted for Model 2 variables plus BMI, eGFR, hypertension, diabetes, hyperlipidemia, and NAFLD.

Note: For each fibrosis score, variables used in the calculation of that score were not additionally included in the corresponding multivariable model, as described in the Methods.

APRI, aspartate aminotransferase to platelet ratio index score; BMI, body mass index; CI, confidence interval; CKM, cardiovascular-kidney-metabolic; eGFR, estimated glomerular filtration rate; FIB-4, Fibrosis-4 index score; LiverRisk, LiverRisk score; MAF-5, metabolic dysfunction-associated fibrosis-5 score; NAFLD, nonalcoholic fatty liver disease; NFS, nonalcoholic fatty liver disease fibrosis score; PIR, poverty income ratio; SAFE, steatosis-associated fibrosis estimator score; SD, standard deviation; sHR, subdistribution hazard ratio.
